# Supplementary material for: The western redcedar genome reveals low genetic diversity in a self-compatible conifer
Source: Genome Res. 2022 Oct;32(10):1952–64. doi: 10.1101/gr.276358.121 (PMC9712635; doi:10.1101/gr.276358.121)
Supplement: Supplemental Material [file supp_32_10_1952__DC1.html]

The western redcedar genome reveals low genetic diversity in a self-compatible conifer — Supplemental Material 

# The western redcedar genome reveals low genetic diversity in a self-compatible conifer

## Supplemental Material

- Supplemental\_Code1\_maps.tar.gz
- Supplemental\_Code2\_differen.tar.gz
- Supplemental\_Code3\_plots.tar.gz
- Supplemental\_Code4\_analysis.tar.gz
- Supplemental\_Code5\_correct.tar.gz
- Supplemental\_Code6\_heter.tar.gz
- Supplemental\_Code7\_coeff.tar.gz
- Supplemental\_Code8\_annot.tar.gz
- Supplemental\_Dataset1.fa.gz
- Supplemental\_Dataset2.xlsx
- Supplemental\_Dataset3.txt
- Supplemental\_Dataset4.fasta
- Supplemental\_Dataset5.vcf.gz
- Supplemental\_Dataset6\_snps.xlsx
- Supplemental\_Dataset7.csv
- Supplemental\_Dataset8.tar.gz
- Supplemental\_Dataset9.csv
- Supplemental\_Dataset10.csv
- Supplemental\_Information.pdf
