# Supplementary material for: The western redcedar genome reveals low genetic diversity in a self-compatible conifer
Source: Genome Res. 2022 Oct;32(10):1952–64. doi: 10.1101/gr.276358.121 (PMC9712635; doi:10.1101/gr.276358.121)
Supplement: Supplemental Material [file supp_gr.276358.121_Supplemental_Dataset4.fasta.html]

Supplemental\_Dataset4 

# The western redcedar genome reveals low genetic diversity in a self-compatible conifer
