# Supplementary material for: The western redcedar genome reveals low genetic diversity in a self-compatible conifer
Source: Genome Res. 2022 Oct;32(10):1952–64. doi: 10.1101/gr.276358.121 (PMC9712635; doi:10.1101/gr.276358.121)
Supplement: Supplemental Material [file supp_gr.276358.121_Supplemental_Information_.pdf]

Supplemental Information for

The western redcedar genome reveals low genetic diversity in a self-compatible conifer

Tal J. Shalev, Omnia Gamal El-Dien, Macaire M.S. Yuen, Shu Shengqiang, Shaun D. Jackman, René L. Warren, Lauren Coombe, Lise van der Merwe, Ada Stewart, Lori B. Boston, Christopher Plott, Jerry Jenkins, Guifen He, Juying Yan, Mi Yan, Jie Guo, Jesse W. Breinholt, Leandro G. Neves, Jane Grimwood, Loren H. Rieseberg, Jeremy Schmutz, Inanc Birol, Matias Kirst, Alvin D. Yanchuk, Carol Ritland, John H. Russell, Joerg Bohlmann

*Corresponding authors: Tal J. Shalev and Joerg Bohlmann.
Email: tal.shalev@msl.ubc.ca; bohlmann@msl.ubc.ca

This PDF file includes:

Supplemental Methods
Figures S1 to S9
Tables S1 to S15
Summaries for Datasets S1 to S10
Summaries for Code S1 to S8
SI References

Other supplementary materials for this manuscript include the following:

Datasets S1 to S10
Code S1 to S8

Supplemental Methods

DNA extraction for genome sequencing and SNP genotyping

Foliar tissue was used for DNA extraction for genome sequencing and genotyping. For genome sequencing, purified nuclear genomic DNA was extracted at BioS&T (http://www.biost.com/, Montreal, Canada) (Birol et al. 2013) and sequenced at the Joint Genome Institute (JGI; Berkeley, USA). For SNP genotyping, DNA was isolated from lyophilized tissue with a modified protocol of Xin and Chen (Xin and Chen 2012). DNA extraction protocol modifications included: the addition of 10% polyvinylpyrrolidone to the CTAB extraction buffer, all centrifugation steps were performed for 10-12 min at 10,000 rpm, the amount of Qiagen MagAttract solution was increased to 7µl, one-minute wait time for binding on the magnetic block was added, and 115µl TE buffer was used for DNA dilution. The concentration and quality of DNA was verified with a Nanodrop 2000c (Fisher Scientific, Toronto, ON, Canada), Quantiflor (Promega Corporate, Madison, WI, USA) and 0.8% agarose gel to cross check for the quantity and quality of the DNA.

Genome sequencing and assembly

Genome sequencing was executed using three types of libraries: short fragment paired-end, large fragment mate-pair, and linked-reads from large molecules using 10× Genomics Chromium. Illumina paired-end adapters were trimmed using Trimadap v0.1r11 (https://github.com/lh3/trimadap). Mate-pair sequencing adapters were removed using NxTrim v0.4.3 (O'Connell et al. 2015). For 10× Genomics libraries, adapters were trimmed using Long Ranger Basic v2.1.6 (https://github.com/10XGenomics/longranger). Depth of *k*-mer coverage

profiles were computed for multiple values of *k* using ntCard v1.0.1 (Mohamadi et al. 2017) (**Figure S8**). The largest value of *k* providing a *k*-mer coverage of at least 15 was selected, based on an estimated coverage of > 99.9%, yielding *k* = 128 (Lander and Waterman 1988). We analyzed and visualized *k*-mer profiles using GenomeScope v1.0.0 (Vurture et al. 2017). Paired-end reads were assembled using ABySS v2.1.4 (parameters: k=128; kc=3) and scaffolded using the mate-pair reads with ABySS-Scaffold (Jackman et al. 2017) (**Figure S9**). Linked-reads were aligned and misassemblies were identified and corrected with Tigmint v1.1.2 (Jackman et al. 2018). The assembly was scaffolded using the linked-reads with ARCS v1.0.5 (Yeo et al. 2018) (-c 2; -m 4-20000) and ABySS-Scaffold (-n 5-7; -s 5000-20000). Molecule size of the linked read libraries was estimated using ChromeQC v1.0.4 (https://bcgsc.github.io/chromeqc). Assembled scaffolds were screened against bacterial proteins, organelle (chloroplast and mitochondria) sequences, and adapters on submission to NCBI and contaminants were removed.

Genome annotation

RNA extraction

Young and mature foliage, xylem and bark samples from a variety of samples (NCBI Umbrella BioProject NCBI PRJNA704616) were ground to a fine powder and total RNA extracted using Purelink ®Plant RNAReagent (Life Technologies, Carlsbad, CA, USA). RNA concentration was determined using a NanoDrop 1000 (Thermo Scientific, Waltham, MA, USA). RNA integrity (RIN) was assessed using Bioanalyzer 2100 RNA Nano chip assays (Agilent, Santa Clara, CA, USA). The minimum RIN was 8.

Transcriptome sequencing and gene space annotation

For PacBio Iso-Seq, full-length cDNAs were synthesized from total RNA using template-switching technology with SMARTer PCR cDNA Synthesis kit (Clontech, CA, USA). First-strand cDNA was amplified with PrimeSTAR GL DNA Polymerase (Clontech) using template-switching oligos to produce double-stranded cDNA, which was purified with AMPure PB beads and either non-size selected or selected for 2-10 kb cutoff by BluePippin (Sage Science, MA, USA). Amplified cDNA was end-repaired and ligated with blunt end PacBio sequencing adaptors using SMRTbell Template Prep Kit 1.0 (Pacific Biosciences, CA, USA), exonuclease treated to remove unligated products, and purified using AMPure PB beads. PacBio Sequencing primer was annealed to the SMRTbell template library and sequencing polymerase was bound using Sequel II Binding kit 1.0. SMRTbell template libraries were sequenced on a Pacific Biosciences' Sequel II sequencer using v4 sequencing primer, 8M v1 SMRT cells, and v1.0 sequencing chemistry with 1×1440 (24-hour) sequencing run times. For Illumina RNA-seq libraries, sample prep was performed on the PerkinElmer Sciclone NGS robotic liquid handling system using Illumina TruSeq Stranded mRNA HT sample prep kit utilizing poly-A mRNA selection (https://support.illumina.com/sequencing/sequencing_kits/truseq-stranded-mrna.html). Libraries were quantified using KAPA Biosystem's next-generation sequencing library qPCR kit and run on a Roche LightCycler 480 real-time PCR instrument. Libraries were multiplexed and the pool of libraries was prepared for sequencing on the Illumina NovaSeq 6000 sequencing platform using NovaSeq XP v1 reagent kits, S4 flow cell, following a 2×150 indexed run recipe.

Gene space annotation was carried out using transcript assemblies from 1) 1.4B 2×150 and 50M 2×100 stranded paired-end Illumina RNA-seq reads using PERTRAN (Shengqiang et al. 2013); 2) 18M PacBio Iso-Seq Circular Consensus Sequences (CCS) which were corrected and collapsed by genome guided correction pipeline to obtain 530K putative full-length

transcripts; and 3) previous RNA-seq assemblies (Shalev et al. 2018). PASA v2.0.2 (Haas et al. 2003) was used to assemble 375,524 transcripts from these three sources. Briefly, PERTRAN executes genome-guided transcriptome short-read (Illumina) assembly via GSNAP (Wu and Nacu 2010) and builds splice alignment graphs after alignment validation, realignment and correction; Iso-Seq CCS correction and clustering aligns CCS reads to the genome with GMAP (Wu and Nacu 2010) while performing intron correction for small indels in splice junctions, if present, and clusters alignments when all introns are the same or have at least 95% overlap for single exon. Gene loci were determined by transcript assembly alignments and EXONERATE v2.4.0 (Slater and Birney 2005) alignments of proteins from *Arabidopsis thaliana*, *Glycine max*, *Populus trichocarpa*, *Oryza sativa*, *Vitis vinifera*, *Aquilegia coerulea*, *Solanum lycopersicum*, *Amborella trichopoda*, *Physcomitrella patens*, *Selaginella moellendorffii*, *Sphagnum magellanicum*, UniProt Pinales and Cupressales, and Swiss-Prot proteomes to the repeat-soft-masked WRC genome using RepeatMasker v4.0.8 (Smit et al. 2015) with up to 2 kbp extension on both ends unless extending into another locus on the same strand. For EXONERATE alignments, an intron size cut-off of 90 kb was used. The repeat library consisted of *de novo* repeats identified by RepeatModeler v1.0.11 (Smit and Hubley 2015) from the WRC genome and repeats in RepBase. Gene models were predicted by homology-based predictors, FGENESH+ v3.1.1 (Salamov and Solovyev 2000), FGENESH_EST v2.6, and EXONERATE and PASA assembled ORFs (in-house homology constrained ORF finder). The best-scored predictions for each locus were selected using multiple positive factors, including transcriptome, EST and protein support, and one negative factor; overlap with repeats. The selected gene predictions were improved by PASA, adding untranslated regions (UTRs), splicing correction, and alternative transcripts. PASA-improved gene models were subjected to protein homology

analysis to the proteomes mentioned above to obtain Cscore (BLASTP score ratio to mutual best hit BLASTP score) and protein coverage (highest percentage of protein aligned to the best homologs). PASA-improved transcripts were selected based on the Cscore, protein coverage, transcriptome and EST coverage, and the overlap of coding sequence (CDS) with repeats. Transcripts were selected with Cscore ≥ 0.5 and protein coverage ≥ 0.5, or if they had transcriptome or EST coverage, but their CDS overlap with repeats was less than 20%. For gene models with CDS overlap with repeats of more than 20%, Cscore was required to be at least 0.9 and homology coverage at least 70% to be selected. Selected gene models were subjected to Pfam analysis. Gene models > 30% of the protein in Pfam TE domains were removed, along with weak gene models (HMMER e-value < 10^{-5}). Incomplete gene models, low homology support without fully transcriptome supported gene models and short single exons (< 300 bp CDS) without protein domains nor strong expression gene models were manually removed. All software was run using default parameters.

SNP genotyping

Targeted sequencing-based genotyping was done by Capture-Seq methodology at Rapid Genomics (Gainesville FL, USA). A set of 57,000 probes was designed for initial marker discovery, from which a panel of 20,858 probes was selected for genotyping. A set of transcriptomes (Shalev et al. 2018) (PRJNA704616) was aligned to the reference genome to identify SNPs.

Candidate probes (120 nt) were initially designed *in silico* and were selected by removing candidates with poor base composition for hybridization (GC content <0.2 and >0.6, high G content >0.2 and long homopolymers >7), followed by removing probes aligning to more than

one position on the reference genome (≥90% identity and length). A final set of 57,000 probes was selected, representing 14,517 scaffolds (average 3.9 probes/scaffold), with 37,294 targeting at least one SNP and 19,706 mapping to intergenic regions not containing pre-identified SNPs.

A set of 128 individuals were selected to validate the probe panel and associated polymorphisms. Genomic DNA (0.5 μg) was fragmented (mean size 300 bp), followed by repair of ends, phosphorylation, adenylation, ligation of Illumina compatible adapters containing 8bp indexes and 5' T-overhang, and 10 cycles PCR amplification with universal primers to produce sequencing-ready libraries. Libraries were quantified using PicoGreen. Libraries from 16 samples were pooled, hybridized to the 120 nt RNA probes following Agilent's SureSelect Target Enrichment System (Agilent Technologies) and sequenced on an Illumina HiSeq X machine with paired-end 150 bp cycle for an average sequencing depth per sample of 15×. Sequence data were aligned to the reference genome with BWA-MEM v0.7.17 (Li 2013) and sets of four samples were combined to increase sequencing depth for identifying markers.

Putative SNPs were identified using FreeBayes v1.2.0 (Garrison and Marth 2012) in 150bp on either side of the 57,000 probes and filtered probes that had more than 17 SNPs per 420 bp target region (150 bp + 120 bp + 150 bp) to avoid over-capture. The sequencing depth of the probes was used to select the final set of 20,858 probes, removing probes on both sides of the distribution (low and high sequencing depth), for Capture-Seq on the remainder of the samples.

SNP discovery was carried out for a large group of 4,832 trees, which included our range-wide population (RWP), selfing lines (SLs), and training and target populations for genomic selection; the latter two were not included in analyses for this study.

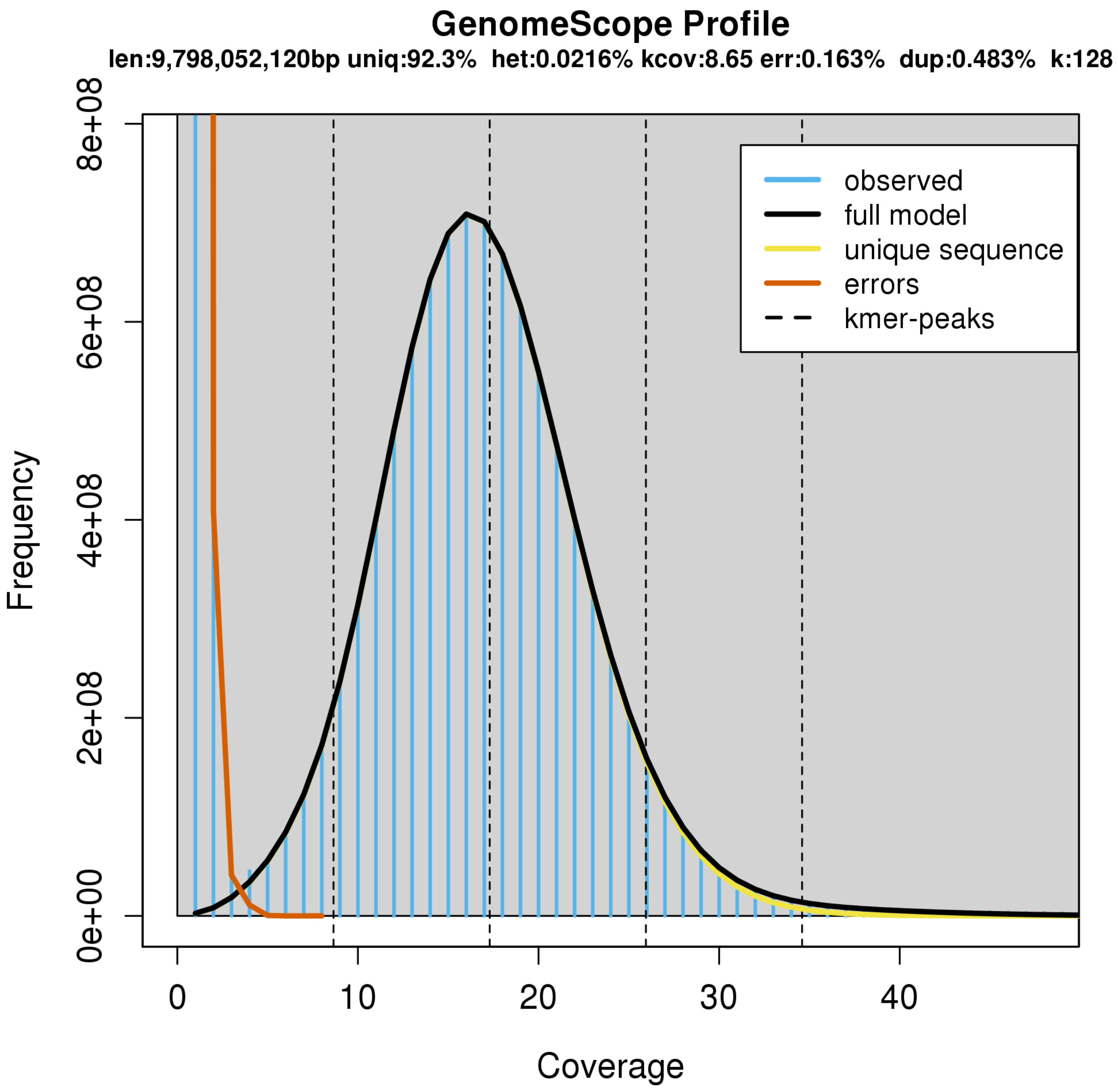

Figure S1. GenomeScope (Vurture et al. 2017) **estimation of western redcedar (WRC) genome size.** Genomescope estimated the size of the WRC genome at 9.8 Gbp.

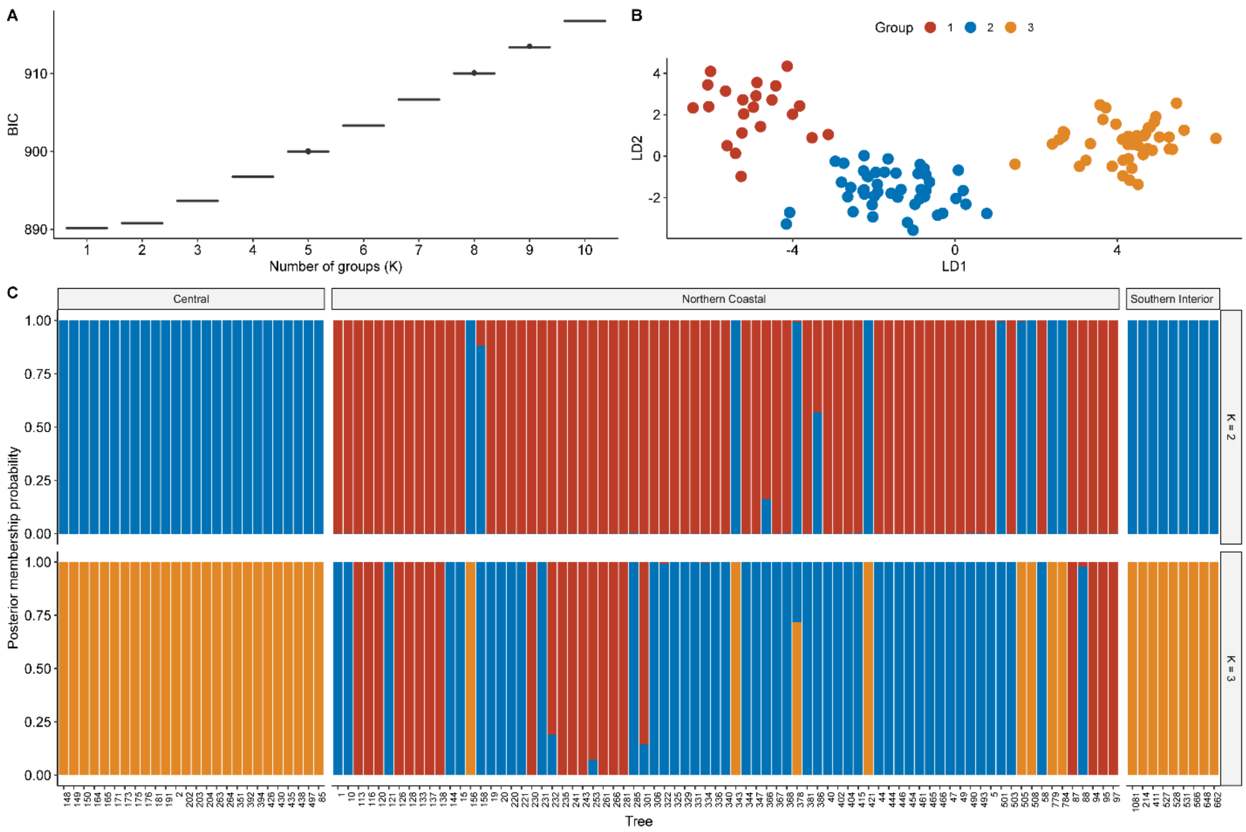

Figure S2. Discriminant Analysis of Principal Components (DAPC) assessment of optimal K for trees in the range-wide population (RWP) (*n* = 112). A) Selection of optimal K using the find.clusters approach. Optimal K is typically determined by selecting the K with lowest Bayesian Information Criterion (BIC) score. However, when the lowest BIC is assigned to K = 1, this is interpreted as an inability to resolve clusters (Miller et al. 2020). **B) DAPC clustering using the first 22 principal components and K = 3. C) Composition plots of the reassignment of individual trees to clusters based on DAPC for K = 2-3.** DAPC was not able to properly reassign trees from the RWP, placing all trees from the Central and Southern-Interior subpopulations together while showing complete mixing in the Northern-Coastal subpopulation.

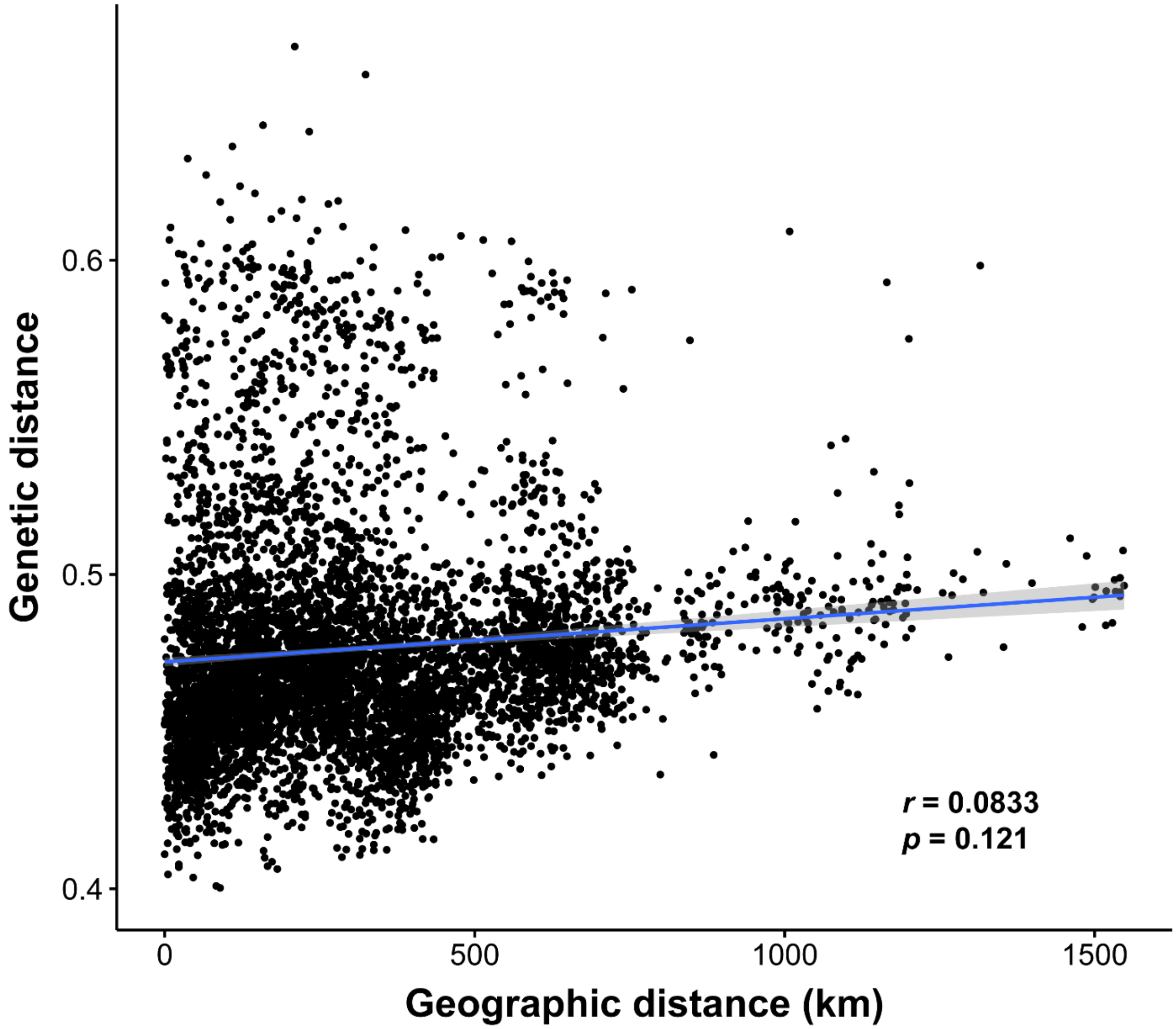

Figure S3. Individual genetic distance as a factor of geographic distance in the range-wide population (n = 112). No significant association was observed between geographic distance and genetic distance when grouping trees based on geographic origin. Surprisingly, no significant association was observed between individual trees either (as shown in the figure), which would normally be expected in continuous populations expanding their range from one source.

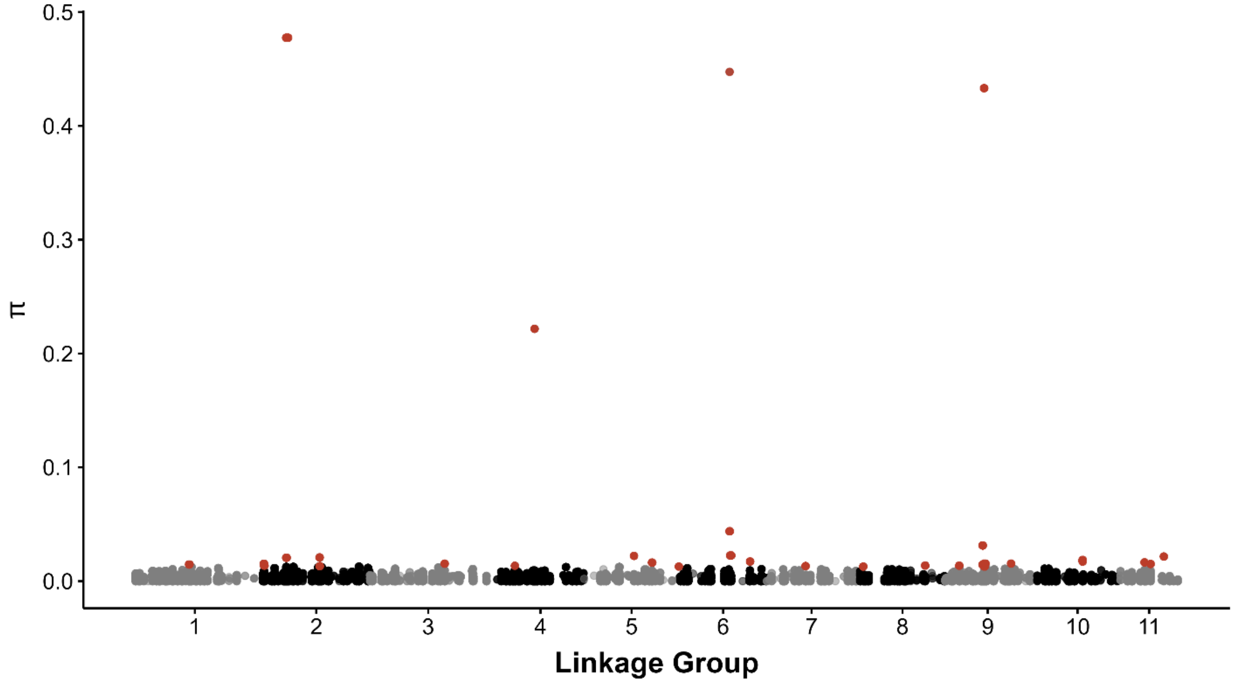

Figure S4. Nucleotide diversity (π) in the range-wide population (*n* = 112). Average π in Single Copy Ortholog (SCO) genes across each putative linkage group (see **Methods and Materials**). The top 1% of SCOs with highest π, which account for 3% of total nucleotide diversity, are shown in red (*n* = 47; **Table S10**).

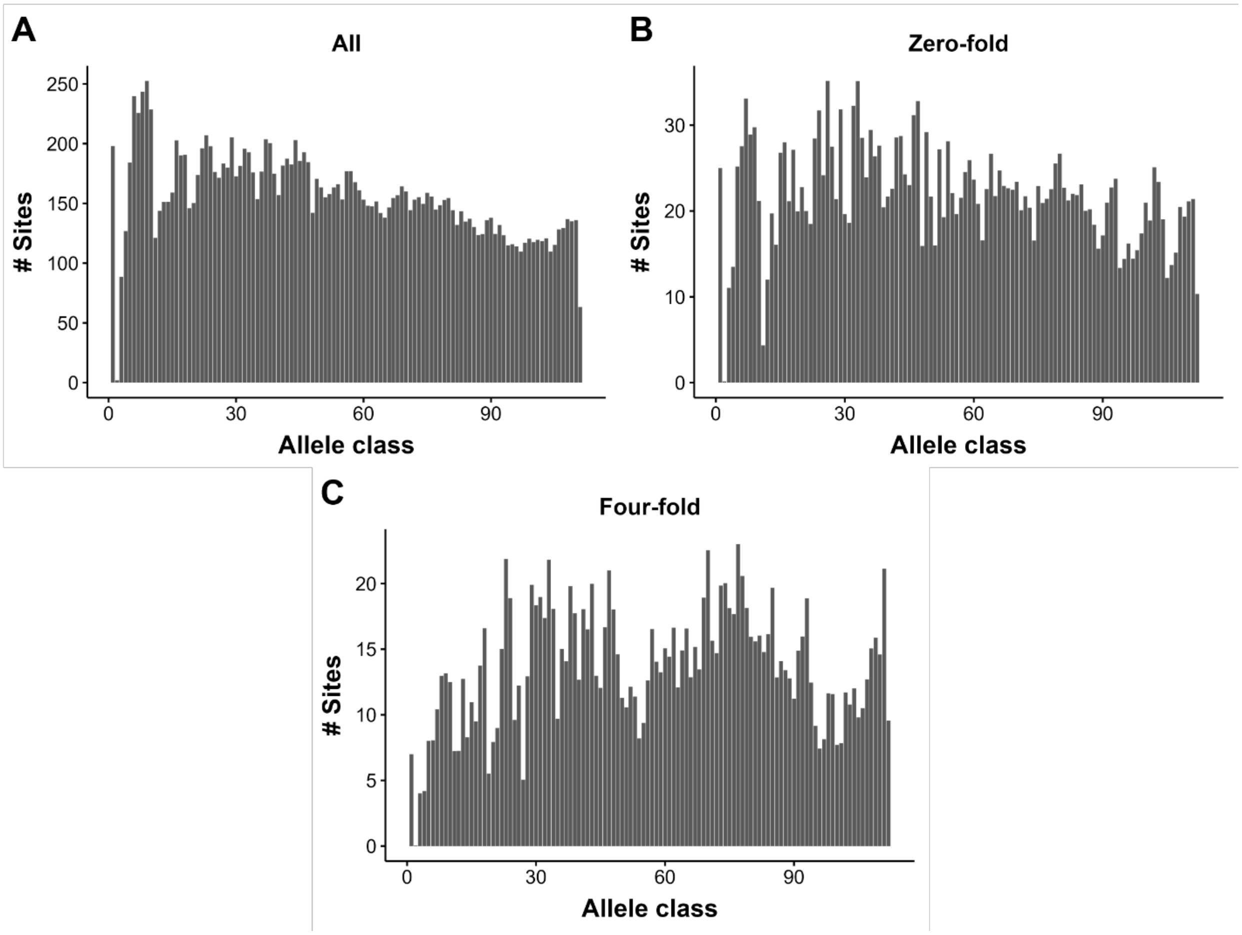

Figure S5. Folded Site Frequency Spectrum (SFS) plots for A) All SNPs in the range-wide population (*n* = 18,371); **B)** All zero-fold degenerate SNPs (*n* = 2,674); and **C)** All four-fold degenerate SNPs (*n* = 1,651s).

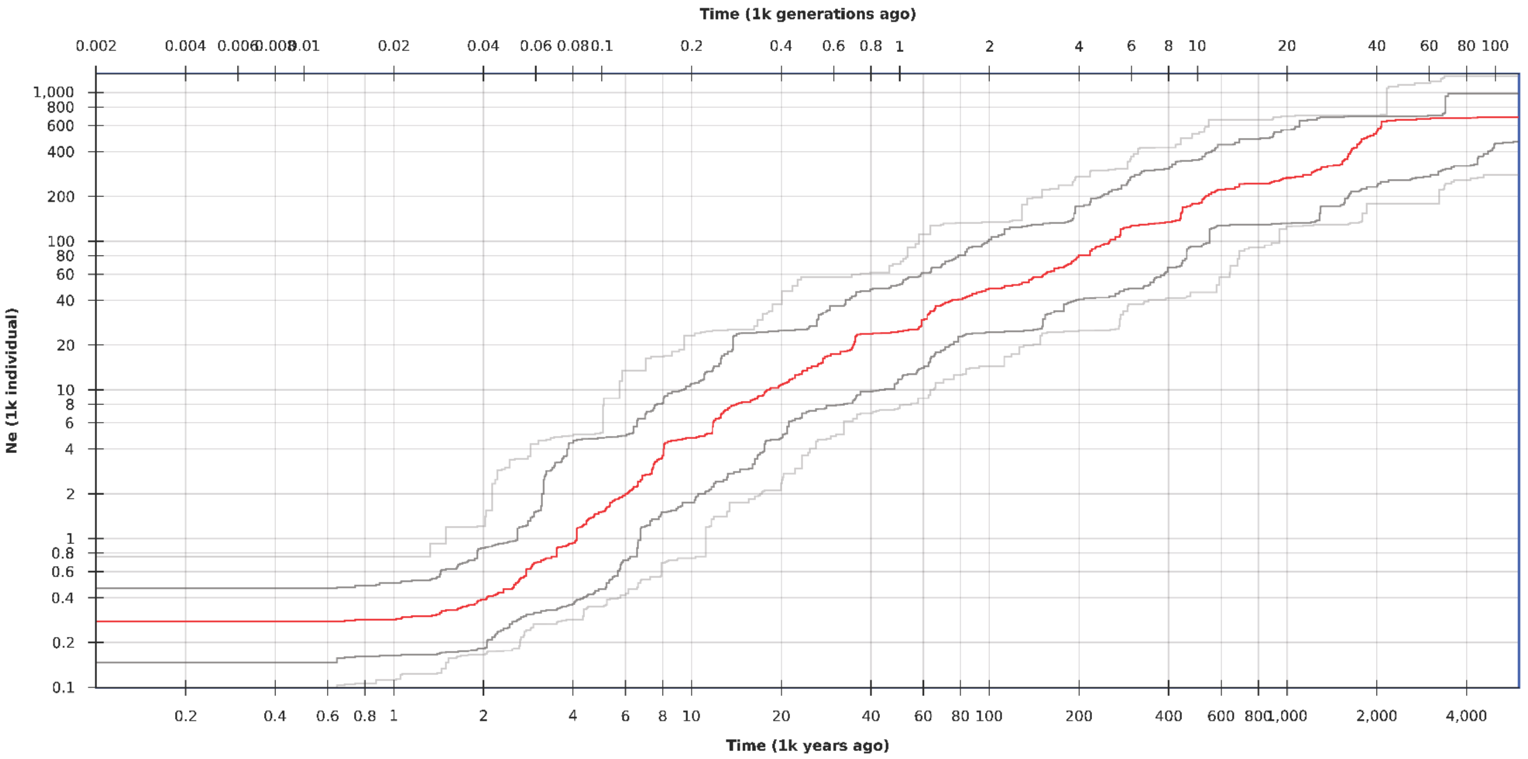

Figure S6. Stairway Plot (Liu and Fu 2020) on folded SFS of intergenic and four-fold degenerate SNPs in the range-wide population. N_e appears to enter a period of decline starting ca. 2 MYA. At the peak of the last glacial period ca. 30 KYA N_e had declined to ~ 20,000 individuals, and continued to drop up until it reached its current level of ~ 300 individuals ca. 2 KYA.

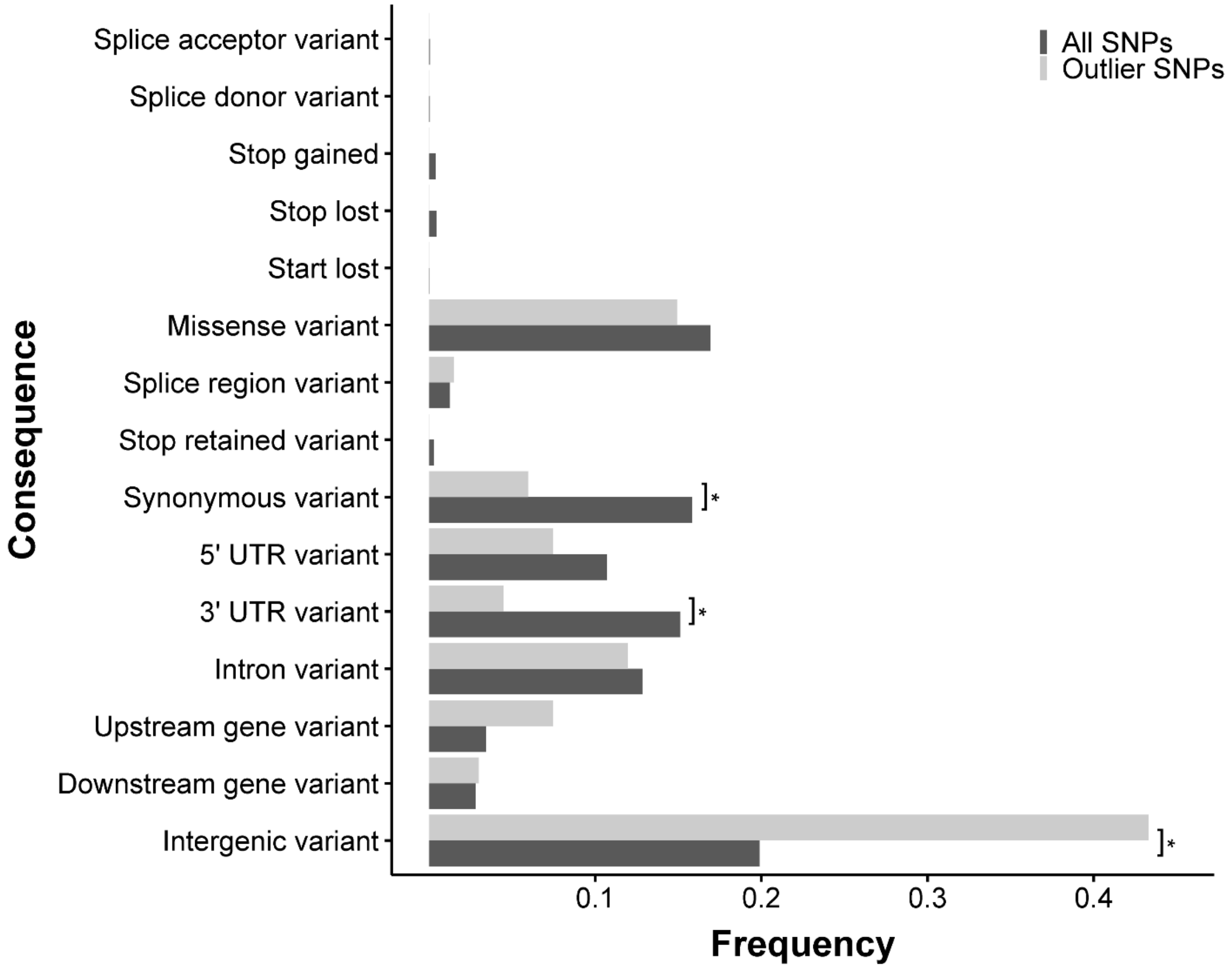

Figure S7. Consequences of SNPs deviating from expectations of genetic drift. Comparison of the consequences of all SNPs with effects predicted by Variant Effect Predictor (VEP) (McLaren et al. 2016) (n = 17,728) and SNPs deviating from expectations of genetic drift with predicted effects (outlier SNPs; n = 83). "*" indicates a significant difference between the two sets of SNPs for proportion of SNPs for a certain consequence (Fisher's Exact Test, $p < 0.05$).

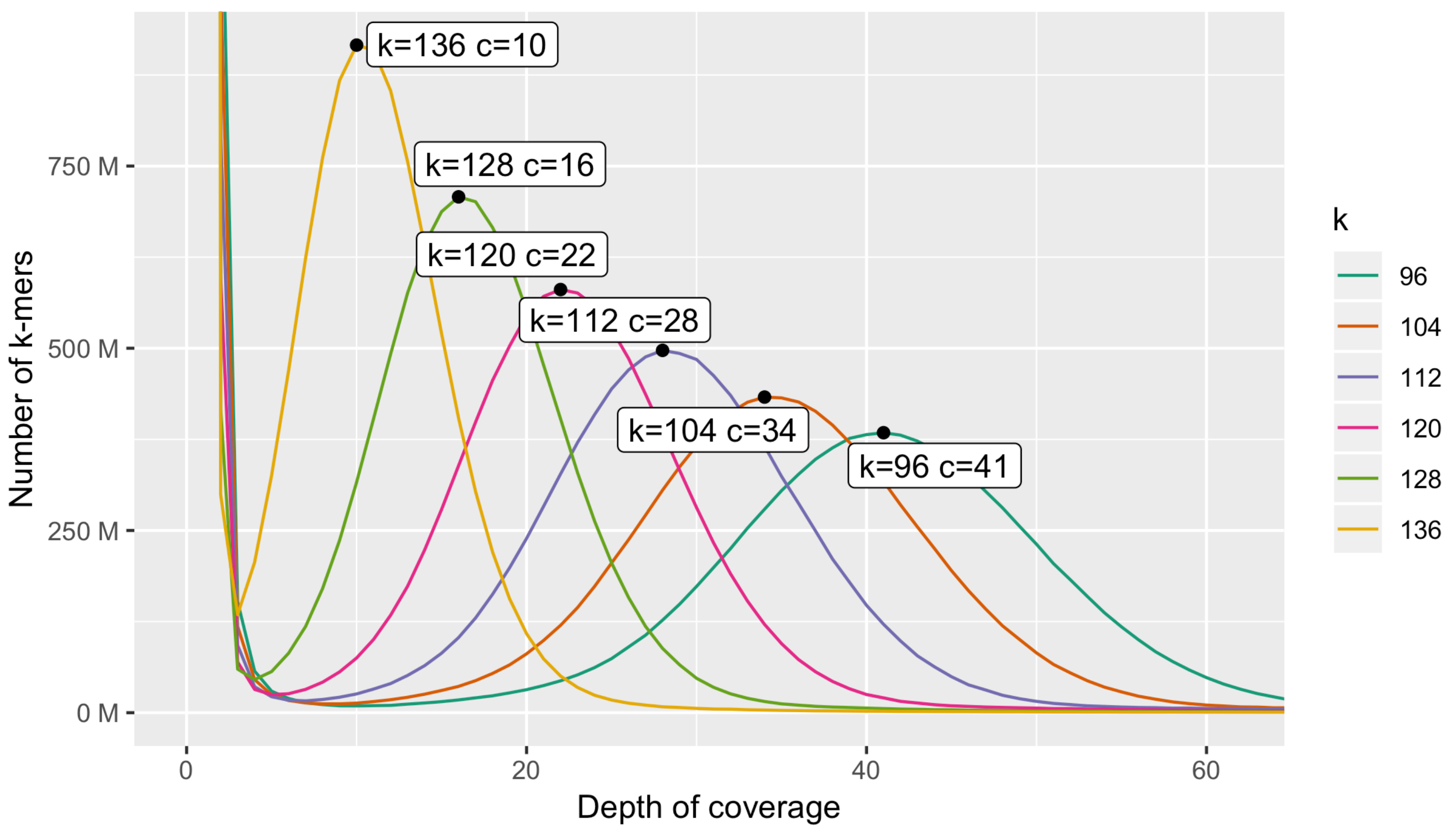

Figure S8. Depth of *k*-mer coverage profiles for multiple values of *k* estimated in ntCard (Mohamadi et al. 2017). Mode of depth of coverage *c* is labeled for each value of *k*.

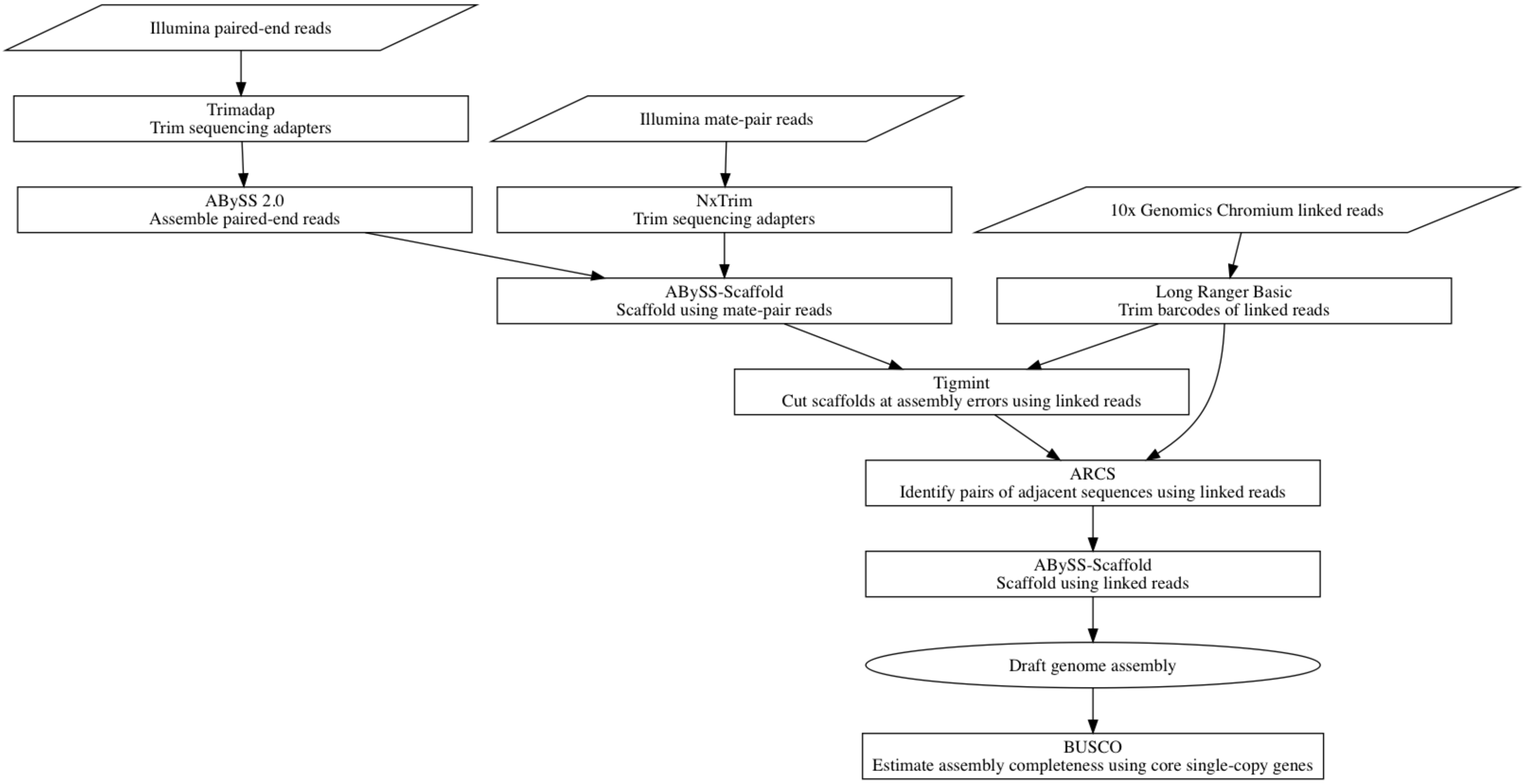

Figure S9. Flowchart of the assembly process of the WRC draft genome.

Table S1. Overview of selfing lines (SLs) selected for the study

	Selfing Line	FS	S1	S2	S3	S4	S5
***	1	1_1	111	111-2	111-22	111-221	111-221-S5
	1	1_2	122	122-1	122-12	122-121	
	1	1_4	145	145-5	145-54	145-545	145-545-S5
***	6	6_1	611	611-1	611-11	611-111	
	6	6_2	623	623-2	623-21	623-211	
***	6	6_4	646	646-4	646-45	646-454	646-454-S5
	7	7_1	712	712-1	712-11	712-111	
***	7	7_2	721	721-2	721-21	721-211	
***	7	7_4	745	745-4	745-44	745-444	
***	8*	8_2	821	821-1	821-11	821-111	
***	8	8_4	845	845-4	845-44	845-444	
	8	8_5	854	854-5	854-54	854-544	
	10	10_1	1011	1011-1	1011-12	1011-121	
***	10	10_2	1022	1022-1	1022-11	1022-113	1022-113-S5
***	10	10_5	1056	1056-4	1056-45	1056-454	
	13	13_2	1323	1323-2	1323-21	1323-211	
***	13	13_3	1332	1332-1	1332-11	1332-111	
***	13	13_4	1345	1345-5	1345-54	1345-544	
***	16	16_1	1611	1611-2	1611-21	1611-211	
	16	16_2	1621	1621-2	1621-22	1621-221	
***	16	16_5	1654	1654-4	1654-45	1654-454	1654-454-S5
***	17	17_2	1721	1721-1	1721-11	1721-111	1721-111-S5
	17	17_4	1744	1744-5	1744-54	1744-544	
***	17	17_5	1755	1755-5	1755-54	1755-545	1755-545-S5
	19	19_1	1912	1912-1	1912-13	1912-131	
***	19	19_2	1922	1922-1	1922-11	1922-111	
***	19	19_5	1955	1955-5	1955-54	1955-544	1955-544-S5
***	20	20_1	2013	2013-1	2013-13	2013-131	
***	20	20_4	2045	2045-5	2045-54	2045-544	
	21	21_1	2111	2111-1	2111-11	2111-111	
***	21	21_2	2121	2121-1	2121-11	2121-111	2121-111-S5
***	21	21_6	2165	2165-4	2165-44	2165-444	2165-444-S5
***	23	23_2	2323	2323-2	2323-21	2323-211	2323-211-S5**
***	23	23_4	2344	2344-4	2344-46	2344-464	

***	26	26_1	2612	2612-1	2612-12	2612-121	
***	26	26_4	2645	2645-4	2645-46	2645-464	
	27	27_1	2712	2712-2	2712-21	2712-211	
***	27	27_2	2721	2721-1	2721-12	2721-121	
	27	27_6	2765	2765-5	2765-55	2765-554	
***	29	29_2	2923	2923-1	2923-12	2923-121	2923-121-S5
***	29	29_4	2944	2944-5	2944-56	2944-565	2944-565-S5

Sequenced; Correctly Labeled
Manually imputed
Sequenced; Mislabeled
Not Sequenced; Dead/Missing
Not Sequenced; not imputed

The first digit is the line identifier. Each subsequent digit identifies the seedling that was chose to continue that SL. Every generation adds an additional digit to assign a unique identifier to each individual up until the S4 generation.

S5 is indicated by adding S5 to the end of the identifier.

* Entire FS line was incorrectly labeled and is unrelated to the other lines starting with "8".
** S5 tree that was used for genome sequencing and assembly.
*** Lines used in heterozygosity and selection under selfing analysis (*n* = 28).

Table S2. Overview of sequencing data generated for the western redcedar draft genome.

Library type	*Fragment*	*Lanes*	*Sequence*	*Depth*
Paired end	431 bp	3	437 Gbp	35
	433 bp	3	412 Gbp	33
	430 bp	2	257 Gbp	20.6
	419 bp	2	252 Gbp	20.2
Total		10	1,358 Gbp	108.6
Mate pair	2.7 kbp	2	239 Gbp	19.1
	5.4 kbp	2	283 Gbp	22.6
	9.0 kbp	2	283 Gbp	22.6
	10.9 kbp	2	282 Gbp	22.6
	15.5 kbp	2	259 Gbp	20.7
Total		10	1,346 Gbp	107.7
Linked-reads	35 kbp	2	236 Gbp	18.9
	35 kbp	2	241 Gbp	19.3
	20 kbp	2	241 Gbp	19.3
	22 kbp	2	241 Gbp	19.3
Total		8	959 Gbp	76.7

The sequencing read length is 2×151 bp. Depth of coverage is calculated using raw sequencing data before trimming adapters and an estimated genome size of 12.8 Gbp.

Table S3. Metrics for each stage of the western redcedar draft genome assembly.

Stage	N50 (Mbp)	NG50 (Mbp)	Largest scaffold (Mbp)	Size (Gbp)	L50 (bp)	LG50 (bp)	Scaffolds
**Unitigs**	0.00698	0.00420	0.0119	7.52	294,835	524,000	1,811,335
**Contigs (PE)**	0.0178	0.0129	166	7.97	128,893	196,169	882,457
**Scaffolds (MP)**	0.377	0.280	2.77	7.95	6,320	9,479	95,754
**Tigmint**	0.375	0.278	2.77	7.95	6,355	9,531	96,557
**ARCS**	2.04	1.50	16.3	7.95	1,176	1,761	68,083
**Tigmint + ARCS**	2.31	1.71	16.3	7.95	1,035	1,551	67,895

NG50 is calculated using a genome size of 10 Gbp, rounded up from the 9.8 Gbp estimated by GenomeScope.

Number of scaffolds and draft genome size includes sequences that are 1 kbp or larger.

"Scaffolds (MP)" shows the assembly contiguity after scaffolding with Illumina mate-pair sequencing.

"Tigmint + ARCS" shows the assembly contiguity after correcting misassemblies using Tigmint and then scaffolding with ARCS. "ARCS" scaffolds the assembly without first correcting misassemblies using Tigmint. Comparing these two rows shows the effects of Tigmint on improving contiguity.

Table S4. A) Genome assembly completeness of seven currently available conifer genome assemblies as estimated by BUSCO v5.0.0 in genome mode (Embryophyta OrthoDB v10, Metaeuk). B) Predicted gene set completeness of seven sequenced conifer genomes as estimated by BUSCO v5.0.0 in protein mode (Embryophyta OrthoDB v10).

A

Taxon	***Thuja plicata***	***Sequoiadendron giganteum*** (Scott et al. 2020)	***Picea glauca*** (Warren et al. 2015)	***Picea abies*** (Nystedt et al. 2013)	***Pinus lambertiana*** (Stevens et al. 2016)	***Pinus taeda*** (Zimin et al. 2014)	***Pseudotsuga menziesii*** (Neale et al. 2017)
	**Western redcedar**	**Giant sequoia**	**White spruce**	**Norway spruce**	**Sugar pine**	**Loblolly pine**	**Douglas- fir**
Family	Cupressaceae	Cupressaceae	Pinaceae	Pinaceae	Pinaceae	Pinaceae	Pinaceae
Complete BUSCOs (C)	1388	1361	802	564	995	803	1196
Complete and single-copy BUSCOs (S)	1293	1272	678	468	881	664	1067
Complete and duplicated BUSCOs (D)	95	89	124	96	114	139	129
Fragmented BUSCOs (F)	128	116	387	433	321	454	242
Missing BUSCOs (M)	98	137	425	617	298	357	176
Total BUSCO groups searched	1614	1614	1614	1614	1614	1614	1614
Percentage complete	86.0%	84.3%	49.7%	34.9%	61.5%	49.7%	74.1%

B

Taxon	***Thuja plicata***	***Sequoiadendron giganteum***	***Picea glauca***	***Picea abies***	***Pinus lambertiana***	***Pinus taeda***	***Pseudotsuga menziesii***
	**Western redcedar**	**Giant sequoia**	**White spruce**	**Norway spruce**	**Sugar pine**	**Loblolly pine**	**Douglas- fir**
Family	Cupressaceae	Cupressaceae	Pinaceae	Pinaceae	Pinaceae	Pinaceae	Pinaceae
Complete BUSCOs (C)	1461	806	291	454	1183	673	1106
Complete and single-copy BUSCOs (S)	1367	747	141	381	1090	579	1014
Complete and duplicated BUSCOs (D)	94	59	150	73	93	94	92
Fragmented BUSCOs (F)	69	260	172	441	121	313	190
Missing BUSCOs (M)	84	548	1151	719	310	628	318
Total BUSCO groups searched	1614	1614	1614	1614	1614	1614	1614
Percentage complete	90.5%	49.9%	18.0%	28.1%	73.3%	41.7%	68.5%

Table S5. Annotation metrics and statistics for the western redcedar draft genome.

**Primary transcripts (loci)**	39,659
**Alternative transcripts (loci)**	26,150
**Total transcripts (loci)**	65,809
**For primary transcripts:**	
Average number of exons per transcript	4.1
Median exon length (bp)	188
Median intron length (bp)	235
Minimum intron length (bp)	20
Maximum intron length (bp)	148,235
**Gene model support (value is number of gene models):**	
Any EST support	33,377
EST support over 100% of their lengths	31,537
EST support over 95% of their lengths	32,083
EST support over 90% of their lengths	32,352
EST support over 75% of their lengths	32,732
EST support over 50% of their lengths	32,983
Peptide homology coverage of 100%	1,388
Peptide homology coverage of over 95%	13,980
Peptide homology coverage of over 90%	19,506
Peptide homology coverage of over 75%	27,030
Peptide homology coverage of over 50%	31,979
Pfam annotation	25,894
Panther annotation	30,847
KOG annotation	12,416
KEGG Orthology annotation	7,088
E.C. number annotation	11,362

Table S6. Verification of the western redcedar (WRC) draft genome annotation. Of a set of 59 WRC transcripts from NCBI and 33 WRC terpene synthase transcripts, 48 and 15 were reliably identified (>90% BLASTN identity, best match in EXONERATE), respectively, in the WRC draft genome.

	Gene	Genomic scaffold	Sequence source
1	gi|1001017507|gb|KU701356.1| Thuja plicata putative LOV domain-containing protein mRNA, complete cds	29379333	NCBI
2	gi|1241840804|gb|KY214317.1| Thuja plicata putative glycoside hydrolase family 2 protein mRNA, complete cds	29378743	NCBI
3	gi|1242943498|gb|KY233467.1| Thuja plicata putative MNS3 mRNA, complete cds	29381436	NCBI
4	gi|1242943576|gb|KY233506.1| Thuja plicata putative sphingomyelin synthetase family protein mRNA, complete cds	29378174	NCBI
5	gi|1242943687|gb|KY233562.1| Thuja plicata putative SPPA1 mRNA, complete cds	29378649	NCBI
6	gi|1242943771|gb|KY249831.1| Thuja plicata putative ABA hypersensitive 1 mRNA, complete cds	29380247	NCBI
7	gi|1242943809|gb|KY249738.1| Thuja plicata putative ABCG7 mRNA, complete cds	29378727	NCBI
8	gi|1242943845|gb|KY249850.1| Thuja plicata putative ATRRP44A mRNA, complete cds	29382381	NCBI
9	gi|1242944211|gb|KY296273.1| Thuja plicata cyclophilin-like peptidyl-prolyl cis-trans isomerase family protein mRNA, complete cds	29377565	NCBI
10	gi|1242944351|gb|KY296166.1| Thuja plicata putative OWL1 mRNA, complete cds	29377407	NCBI
11	gi|1248694559|gb|KY314818.1| Thuja plicata ATTRM11 mRNA, complete cds	29380894	NCBI
12	gi|1248694675|gb|KY314876.1| Thuja plicata FG-GAP repeat-containing protein mRNA, complete cds	29377050	NCBI
13	gi|1248694816|gb|KY314948.1| Thuja plicata INO80 mRNA, complete cds	29379961	NCBI
14	gi|1248694885|gb|KY314984.1| Thuja plicata ATCCH1 fatty acid oxygenation upregulated 2 mRNA, complete cds	29377034	NCBI
15	gi|1248694997|gb|KY367630.1| Thuja plicata cytochrome P450 mRNA, complete cds	29382440	NCBI
16	gi|1248695181|gb|KY367685.1| Thuja plicata FGGY family of carbohydrate kinase mRNA, complete cds	29378653	NCBI
17	gi|1248695219|gb|KY367704.1| Thuja plicata importin alpha isoform 9 mRNA, complete cds	29381189	NCBI
18	gi|1248695293|gb|KY367741.1| Thuja plicata HCF145 mRNA, complete cds	29377609	NCBI
19	gi|1248695329|gb|KY367759.1| Thuja plicata AP4B mRNA, complete cds	29377919	NCBI
20	gi|1248695367|gb|KY367796.1| Thuja plicata no exine fromation 1 mRNA, complete cds	29379245	NCBI

**21**	gi|1248695439|gb|KY367851.1| Thuja plicata Spc97 / Spc98 family mRNA, complete cds	29379997	NCBI
**22**	gi|1248695589|gb|KY367926.1| Thuja plicata saccharopine dehydrogenase mRNA, complete cds	29381726	NCBI
**23**	gi|1248695663|gb|KY367963.1| Thuja plicata ATDUR3 mRNA, complete cds	29377833	NCBI
**24**	gi|1248695843|gb|KY368053.1| Thuja plicata Zn-dependent exopeptidases superfamily protein mRNA, complete cds	29382329	NCBI
**25**	gi|1248695921|gb|KY368092.1| Thuja plicata ATDPE1 mRNA, complete cds	29377587	NCBI
**26**	gi|1786237212|gb|MK817116.1| Thuja plicata DXS1 mRNA, complete cds	29379753	NCBI
**27**	gi|1786237229|gb|MK817117.1| Thuja plicata DXS2B mRNA, complete cds	29381756	NCBI
**28**	gi|1786237253|gb|MK817118.1| Thuja plicata DXS2A mRNA, complete cds	29378340	NCBI
**29**	gi|1786237278|gb|MK817119.1| Thuja plicata DXS3 mRNA, complete cds	29377322	NCBI
**30**	gi|1890528724|gb|MT468207.1| Thuja plicata diterpene synthase (diTPS1) mRNA, complete cds	29379660	NCBI
**31**	gi|1890528727|gb|MT468208.1| Thuja plicata diterpene synthase (diTPS2) mRNA, complete cds	29381898	NCBI
**32**	gi|1890528729|gb|MT468209.1| Thuja plicata diterpene synthase (diTPS3) mRNA, complete cds	29382319	NCBI
**33**	gi|510793902|gb|KC767281.1| Thuja plicata sabinene synthase mRNA, complete cds	29377789	NCBI
**34**	gi|635148812|gb|KJ195050.1| Thuja plicata phototropin (PHOT1) mRNA, complete cds	29378985	NCBI
**35**	gi|635148826|gb|KJ195057.1| Thuja plicata phototropin (PHOT2) mRNA, complete cds	29378009	NCBI
**36**	gi|6694696|gb|AF210063.1| Thuja plicata dirigent-like protein (psd_Tp1) mRNA, complete cds	29378666	NCBI
**37**	gi|6694698|gb|AF210064.1| Thuja plicata dirigent protein (psd_Tp2) mRNA, complete cds_Tp4) mRNA, complete cds	29377524	NCBI
**38**	gi|6694702|gb|AF210066.1| Thuja plicata dirigent-like protein (psd_Tp4) mRNA, complete cds	29380546	NCBI
**39**	gi|6694706|gb|AF210068.1| Thuja plicata dirigent-like protein (psd_Tp6) mRNA, complete cds	29377169	NCBI
**40**	gi|6694708|gb|AF210069.1| Thuja plicata dirigent protein (psd_Tp7) mRNA, complete cds	29207864	NCBI
**41**	gi|7542587|gb|AF242506.1| Thuja plicata clone 4 pinoresinol-lariciresinol reductase mRNA, complete cds	29377837	NCBI
**42**	gi|7578912|gb|AF242500.1| Thuja plicata phenylcoumaran benzylic ether reductase homolog Tp1 mRNA, complete cds	29381391	NCBI
**43**	gi|821372382|gb|KP004988.1| Thuja plicata (+)-sabinene-3-hydroxylase mRNA, complete cds	29379924	NCBI
**44**	gi|821372384|gb|KP015848.1| Thuja plicata cytochrome P450 CYP76AA20 mRNA, complete cds	29381501	NCBI
**45**	gi|821372386|gb|KP015849.1| Thuja plicata cytochrome P450 CYP76AA21 mRNA, complete cds	29380148	NCBI

**46**	gi|821372394|gb|KP015853.1| Thuja plicata cytochrome P450 CYP76AA25 mRNA, complete cds	29377693	NCBI
**47**	gi|821372396|gb|KP015854.1| Thuja plicata cytochrome P450 CYP76AA26 mRNA, complete cds	29377042	NCBI
**48**	gi|821372398|gb|KP015855.1| Thuja plicata cytochrome P450 CYP76Z2 mRNA, complete cds	29377249	NCBI
**1**	TpTPS2_evgvelvLoc955t9	29194648	Putative WRC terpene synthase transcripts
**2**	TpTPS3_evgvelvLoc2319t10	29382319	Putative WRC terpene synthase transcripts
**3**	TpTPS10_evgvelvLoc9436t1	29381898	Putative WRC terpene synthase transcripts
**4**	TpTPS13_evgvelvLoc5170t3	29378510	Putative WRC terpene synthase transcripts
**5**	TpTPS21_evgvelvLoc4915t2	29379564	Putative WRC terpene synthase transcripts
**6**	TpTPS24_evgvelvLoc16400d36129t1	29381804	Putative WRC terpene synthase transcripts
**7**	TpTPS25_evgvelvLoc2138t7	29382319	Putative WRC terpene synthase transcripts
**8**	TpTPS26_evgvelvLoc4951t5	29380592	Putative WRC terpene synthase transcripts
**9**	TpTPS27_evgvelvLoc1076t3	29377789	Putative WRC terpene synthase transcripts
**10**	TpTPS28_evgvelvLoc842d111924t1	29379713	Putative WRC terpene synthase transcripts
**11**	TpTPS29_evgvelvLoc7235d121309t1	29380776	Putative WRC terpene synthase transcripts
**12**	TpTPS30_evgvelvLoc16257d162311t1	29381146	Putative WRC terpene synthase transcripts
**13**	TpTPS31_evgR1187289	29380228	Putative WRC terpene synthase transcripts
**14**	TpTPS32_evgR741119	29379604	Putative WRC terpene synthase transcripts
**15**	TpTPS33_evgS303450	29380592	Putative WRC terpene synthase transcripts

Table S7. The range-wide population (*n* = 112) of western redcedar used in this study. The population was stratified into three subpopulations based on the analysis of O'Connell et al. (2008).

Tree ID	Subpopulation	Location	Latitude (° N)	Longitude (° W)	Elevation (m)
**2**	Central	Jordan River	48.477	-123.929	50
**85**	Central	Powell Lake	50.233	-124.22	15
**148**	Central	Tzoonie River	49.867	-123.44	425
**149**	Central	Tzoonie River	49.867	-123.44	365
**150**	Central	Tzoonie River	49.867	-123.733	450
**164**	Central	Powell Lake	50.30	-124.37	15
**165**	Central	Lois Lake	49.817	-124.19	250
**171**	Central	Lewis Lake	49.933	-124.22	470
**173**	Central	Labour Day Lake	49.133	-124.27	975
**175**	Central	Francis Lake	48.933	-124.42	503
**176**	Central	San Mateo Bay	48.95	-124.59	90
**181**	Central	Cameron Lake	49.32	-124.48	15
**191**	Central	Jervis Inlet	50.117	-123.783	98
**202**	Central	Red Tusk	49.767	-123.24	240
**203**	Central	Sechelt Creek	49.676	-123.535	400
**204**	Central	Bear Creek	49.8	-123.32	580
**263**	Central	Narrows Inlet	49.083	-123.733	120
**264**	Central	Narrows Inlet	49.083	-123.44	210
**351**	Central	Second Lake	49.1	-124.13	300
**392**	Central	Hardy Island	49.75	-124.18	37
**394**	Central	Lois Lake	49.8	-124.17	262
**426**	Central	Jordan River	48.52	-124.17	730
**430**	Central	Errington	49.267	-124.05	152
**435**	Central	Chemainus	48.933	-123.75	115
**438**	Central	Bamberton	48.617	-123.33	168
**497**	Central	Caycuse Lake	48.8	-124.36	230
**1**	Northern-Coastal	Keogh River	50.617	-127.19	61
**5**	Northern-Coastal	Mahatta Creek	50.4	-127.783	122
**10**	Northern-Coastal	Mahatta Creek	50.317	-127.39	396
**15**	Northern-Coastal	Mahatta Creek	50.4	-127.783	155
**19**	Northern-Coastal	Benson Lake	50.4	-127.26	637
**20**	Northern-Coastal	Snowsaddle Mountain	50.25	-127.33	305
**40**	Northern-Coastal	Keogh River	50.633	-127.23	91
**44**	Northern-Coastal	Waukwaas Creek	50.55	-127.18	183
**47**	Northern-Coastal	Waukwaas Creek	50.583	-127.21	30

**49**	Northern-Coastal	Misty Lake	50.633	-127.283	91
**58**	Northern-Coastal	MacJack River	50.617	-128.09	60
**87**	Northern-Coastal	Louise Island	52.92	-131.85	600
**88**	Northern-Coastal	Skedans Islands	52.95	-131.57	395
**94**	Northern-Coastal	Talunkwan Island	52.829	-131.752	335
**95**	Northern-Coastal	Tasu	52.817	-132.04	275
**97**	Northern-Coastal	Tasu	52.817	-132.04	565
**113**	Northern-Coastal	Gogit Passage	52.633	-131.29	245
**116**	Northern-Coastal	Sedgwick Bay	52.6	-131.34	305
**120**	Northern-Coastal	Tanu Passage	52.767	-131.44	365
**121**	Northern-Coastal	Tanu Island	52.767	-131.41	305
**126**	Northern-Coastal	Ramsay Island	52.564	-131.379	150
**128**	Northern-Coastal	Huxley Island	52.432	-131.268	185
**133**	Northern-Coastal	Huston Inlet	52.267	-131.17	185
**137**	Northern-Coastal	Tangil Peninsula	52.791	-131.705	305
**138**	Northern-Coastal	Talunkwan Island	52.829	-131.692	215
**144**	Northern-Coastal	Stewardson Inlet	49.417	-126.18	410
**156**	Northern-Coastal	Waring Creek	49.95	-126.08	350
**158**	Northern-Coastal	Cougar Creek	49.717	-126.25	355
**220**	Northern-Coastal	Roselle Lake	50.517	-127	120
**221**	Northern-Coastal	Nimpkish Lake	50.483	-126.59	75
**230**	Northern-Coastal	Long Lake	51.3	-127.02	317
**231**	Northern-Coastal	Long Lake	51.233	-127.14	268
**232**	Northern-Coastal	Naysash Inlet	51.3	-127.2	110
**235**	Northern-Coastal	Amback Creek	51.717	-127.02	189
**241**	Northern-Coastal	Rivers Inlet	51.60	-127.58	300
**243**	Northern-Coastal	Hardy Inlet	51.717	-127.38	159
**253**	Northern-Coastal	Kwatna	52.033	-127.25	985
**261**	Northern-Coastal	Link Lake	52.433	-127.42	950
**266**	Northern-Coastal	Slim Creek	52.87	-132.08	75
**281**	Northern-Coastal	Gwaii Haanas	52.45	-131.45	45
**285**	Northern-Coastal	Misty Lake	50.616	-127.24	90
**301**	Northern-Coastal	Waukwaas River	50.6	-127.23	100
**306**	Northern-Coastal	Denad Creek	50.55	-128	25
**322**	Northern-Coastal	Koprino River	50.55	-127.5	280
**325**	Northern-Coastal	Hathaway Creek	50.55	-127.51	95
**329**	Northern-Coastal	Victoria Lake	50.43	-127.40	123
**331**	Northern-Coastal	Victoria Lake	50.317	-127.22	200
**334**	Northern-Coastal	Snowsaddle Mountain	50.25	-127.2	213
**336**	Northern-Coastal	Victoria Lake	50.35	-127.22	122
**340**	Northern-Coastal	Utlah Creek	50.3	-127.26	183

**343**	Northern-Coastal	Victoria Lake	50.05	-127.417	457
**344**	Northern-Coastal	Victoria Lake	50.05	-127.417	457
**347**	Northern-Coastal	Utlah Creek	50.283	-127.25	244
**366**	Northern-Coastal	Henderson Lake	49.1	-125.02	300
**367**	Northern-Coastal	Henderson Lake	49.1	-125.017	200
**368**	Northern-Coastal	Henderson Lake	49.10	-125.03	280
**378**	Northern-Coastal	Tranquil Inlet	49.2	-125.4	3
**381**	Northern-Coastal	Sydney Inlet	49.483	-126.283	65
**386**	Northern-Coastal	Cypre River	49.367	-125.867	180
**402**	Northern-Coastal	Tahsish River	50.15	-127.13	15
**404**	Northern-Coastal	Chamiss Bay	50.08	-127.35	350
**415**	Northern-Coastal	Lull Creek	50.7	-126.02	183
**421**	Northern-Coastal	Wood Bay	50.317	-125.2	200
**444**	Northern-Coastal	Anutz Lake	50.3	-126.55	80
**446**	Northern-Coastal	Kinman Creek	50.35	-126.54	250
**454**	Northern-Coastal	Mt. Bate	49.85	-126.51	1000
**461**	Northern-Coastal	Artlish Caves	50.133	-127.083	100
**465**	Northern-Coastal	Union Island	50.029	-127.277	50
**466**	Northern-Coastal	Hohoae Island	50.048	-127.207	50
**490**	Northern-Coastal	Mitla Creek	49.5	-125.58	220
**493**	Northern-Coastal	Fortune Creek	49.183	-125.44	365
**501**	Northern-Coastal	Snug Bay	49.033	-125.01	60
**503**	Northern-Coastal	Nahmint Lake	49.183	-125.05	150
**505**	Northern-Coastal	Bigtree Creek	50.267	-125.43	150
**508**	Northern-Coastal	Brasseau Bay	50.4	-125.59	45
**779**	Northern-Coastal	Mt. Washington	49.733	-125.14	950
**784**	Northern-Coastal	Mt. Washington	49.7	-125.05	110
**214**	Southern-Interior	Burke Mountain	49.317	-122.44	440
**411**	Southern-Interior	Debeck Creek	49.483	-122.36	275
**527**	Southern-Interior	Tumtum Lake	51.75	-119.09	960
**528**	Southern-Interior	Tumtum Lake	51.083	-119.09	910
**531**	Southern-Interior	East Barrier Lake	50.85	-119.44	610
**566**	Southern-Interior	Price Creek	40.05	-124.15	50
**648**	Southern-Interior	Rector Ridge	45.783	-123.4	246
**662**	Southern-Interior	Simmons Loop	45.367	-123.45	246
**1081**	Southern-Interior	Alsea River	44.45	-123.59	49

Table S8. SNP effects as predicted by Ensembl Variant Effect Predictor (McLaren et al. 2016) for 17,728 SNPs out of the 18,371 SNPs in our dataset.

SNP effect	# of SNPs	% of total	Region	IMPACT
Splice acceptor variant	9	0.0490	Non-coding	High
Splice donor variant	7	0.0381	Non-coding	High
Stop gained	69	0.376	Coding	High
Stop lost	78	0.425	Coding	High
Start lost	4	0.0218	Coding	High
Missense variant	3002	16.34	Coding	Moderate
Splice region variant	221	1.20	Non-coding	Low
Stop retained variant	50	0.272	Coding	Low
Synonymous variant	2807	15.3	Coding	Low
5' UTR variant	1896	10.3	Non-coding	Modifier
3' UTR variant	2680	14.6	Non-coding	Modifier
Intron variant	2274	12.4	Non-coding	Modifier
Upstream gene variant	607	3.30	Intergenic	Modifier
Downstream gene variant	498	2.71	Intergenic	Modifier
Intergenic variant	3526	19.2	Intergenic	Modifier
Unknown	643	3.50	-	-
**Total**	**18371**			

Effects are listed from top to bottom in order of severity from most to least severe.

IMPACTs are defined by Ensembl as follows:
High: The variant is assumed to have high (disruptive) impact in the protein, probably causing protein truncation, loss of function or triggering nonsense mediated decay.
Moderate: A non-disruptive variant that might change protein effectiveness.
Low: A variant that is assumed to be mostly harmless or unlikely to change protein behaviour.
Modifier: Usually non-coding variants or variants affecting non-coding genes, where predictions are difficult or there is no evidence of impact.

Table S9. Pairwise F_{ST} for all subpopulations. F_{ST} values are based on Weir and Cockerham's estimator (Weir and Cockerham 1984).

Subpopulation	Northern-Coastal	Central	Southern-Interior
**Northern-Coastal**	-	-	-
**Central**	0.0335	-	-
**Southern-Interior**	0.0412	0.00961	-

Table S10. Annotation for single-copy ortholog genes with highest 1% of π estimates of nucleotide diversity (*n* = 47).

Gene	Annotation source	Annotation access code	Annotation
**Thupl.293114653s0001**	PANTHER	PTHR11926:SF500	-
**Thupl.293114653s0001**	KEGGORTH	K08237	hydroquinone glucosyltransferase [EC:2.4.1.218]
**Thupl.293114653s0001**	PANTHER	PTHR11926	GLUCOSYL/GLUCURONOSYL TRANSFERASES
**Thupl.293114653s0001**	GO	GO:0008152	metabolic process
**Thupl.293114653s0001**	GO	GO:0016758	transferase activity, transferring hexosyl groups
**Thupl.293114653s0001**	EC	2.4.1.170	Isoflavone 7-O-glucosyltransferase
**Thupl.293114653s0001**	PFAM	PF00201	UDP-glucoronosyl and UDP-glucosyl transferase
**Thupl.29343737s0001**	GO	GO:0005618	cell wall
**Thupl.29343737s0001**	PFAM	PF04043	Plant invertase/pectin methylesterase inhibitor
**Thupl.29343737s0001**	PANTHER	PTHR31707:SF25	-
**Thupl.29343737s0001**	EC	3.1.1.11	Pectinesterase
**Thupl.29343737s0001**	GO	GO:0004857	enzyme inhibitor activity
**Thupl.29343737s0001**	PFAM	PF01095	Pectinesterase
**Thupl.29343737s0001**	GO	GO:0042545	cell wall modification
**Thupl.29343737s0001**	KEGGORTH	K01051	pectinesterase [EC:3.1.1.11]
**Thupl.29343737s0001**	GO	GO:0030599	pectinesterase activity
**Thupl.29343737s0001**	PANTHER	PTHR31707	FAMILY NOT NAMED
**Thupl.29376925s0001**	KEGGORTH	K08762	diazepam-binding inhibitor (GABA receptor modulator, acyl-CoA-binding protein)
**Thupl.29376925s0001**	PANTHER	PTHR23310	ACYL-COA-BINDING PROTEIN, ACBP
**Thupl.29376925s0001**	PFAM	PF00887	Acyl CoA binding protein
**Thupl.29376925s0001**	GO	GO:0000062	fatty-acyl-CoA binding
**Thupl.29376925s0001**	PANTHER	PTHR23310:SF67	-
**Thupl.29377001s0021**	GO	GO:0016616	oxidoreductase activity, acting on the CH-OH group of donors, NAD or NADP as acceptor
**Thupl.29377001s0021**	KOG	KOG1494	NAD-dependent malate dehydrogenase
**Thupl.29377001s0021**	PFAM	PF00056	lactate/malate dehydrogenase, NAD binding domain
**Thupl.29377001s0021**	PANTHER	PTHR11540:SF21	L-LACTATE DEHYDROGENASE
**Thupl.29377001s0021**	PFAM	PF02866	lactate/malate dehydrogenase, alpha/beta C-terminal domain
**Thupl.29377001s0021**	GO	GO:0055114	oxidation-reduction process
**Thupl.29377001s0021**	GO	GO:0016491	oxidoreductase activity
**Thupl.29377001s0021**	KEGGORTH	K00026	malate dehydrogenase [EC:1.1.1.37]
**Thupl.29377001s0021**	PANTHER	PTHR11540	MALATE AND LACTATE DEHYDROGENASE
**Thupl.29377001s0021**	EC	1.1.1.37	Malate dehydrogenase
**Thupl.29377148s0010**	KOG	KOG3328	HGG motif-containing thioesterase
**Thupl.29377148s0010**	EC	3.1.2.20	Acyl-CoA hydrolase

**Thupl.29377148s0010**	KEGGORTH	K17362	-
**Thupl.29377148s0010**	PANTHER	PTHR21660:SF8	-
**Thupl.29377148s0010**	PANTHER	PTHR21660	THIOESTERASE SUPERFAMILY MEMBER-RELATED
**Thupl.29377148s0010**	PFAM	PF03061	Thioesterase superfamily
**Thupl.29377170s0054**	GO	GO:0045263	proton-transporting ATP synthase complex, coupling factor F(o)
**Thupl.29377170s0054**	GO	GO:0015078	hydrogen ion transmembrane transporter activity
**Thupl.29377170s0054**	KEGGORTH	K02109	F-type H+-transporting ATPase subunit b [EC:3.6.3.14]
**Thupl.29377170s0054**	PANTHER	PTHR33445	-
**Thupl.29377170s0054**	PFAM	PF00430	ATP synthase B/B' CF(0)
**Thupl.29377170s0054**	GO	GO:0015986	ATP synthesis coupled proton transport
**Thupl.29377251s0003**	PANTHER	PTHR33918	-
**Thupl.29377251s0003**	PANTHER	PTHR33918:SF2	-
**Thupl.29377336s0006**	EC	6.3.5.7	Glutaminyl-tRNA synthase (glutamine-hydrolyzing)
**Thupl.29377336s0006**	GO	GO:0016874	ligase activity
**Thupl.29377336s0006**	KOG	KOG2438	Glutamyl-tRNA amidotransferase subunit B
**Thupl.29377336s0006**	PFAM	PF02637	GatB domain
**Thupl.29377336s0006**	PFAM	PF02934	GatB/GatE catalytic domain
**Thupl.29377336s0006**	PANTHER	PTHR11659	GLUTAMYL-TRNA(GLN) AMIDOTRANSFERASE SUBUNIT B (MITOCHONDRIAL AND PROKARYOTIC) PET112-RELATED
**Thupl.29377336s0006**	GO	GO:0016884	carbon-nitrogen ligase activity, with glutamine as amido-N-donor
**Thupl.29377336s0006**	KEGGORTH	K02434	aspartyl-tRNA(Asn)/glutamyl-tRNA (Gln) amidotransferase subunit B [EC:6.3.5.6 6.3.5.7]
**Thupl.29377336s0006**	PANTHER	PTHR11659:SF3	-
**Thupl.29377352s0001**	GO	GO:0005789	endoplasmic reticulum membrane
**Thupl.29377352s0001**	SIGNALP	SignalP-noTM	-
**Thupl.29377352s0001**	PFAM	PF03896	Translocon-associated protein (TRAP), alpha subunit
**Thupl.29377352s0001**	KOG	KOG1631	Translocon-associated complex TRAP, alpha subunit
**Thupl.29377352s0001**	KEGGORTH	K13249	translocon-associated protein subunit alpha
**Thupl.29377352s0001**	PANTHER	PTHR12924	TRANSLOCON-ASSOCIATED PROTEIN, ALPHA SUBUNIT
**Thupl.29377414s0008**	PFAM	PF07145	Ataxin-2 C-terminal region
**Thupl.29377414s0008**	PANTHER	PTHR33790	-
**Thupl.29377534s0013**	PANTHER	PTHR21257	STEROL REDUCTASE/LAMIN B RECEPTOR
**Thupl.29377534s0013**	EC	1.3.1.21	7-dehydrocholesterol reductase
**Thupl.29377534s0013**	GO	GO:0016020	membrane
**Thupl.29377534s0013**	KOG	KOG1435	Sterol reductase/lamin B receptor

**Thupl.29377534s0013**	PFAM	PF01222	Ergosterol biosynthesis ERG4/ERG24 family
**Thupl.29377534s0013**	KEGGORTH	K00213	7-dehydrocholesterol reductase [EC:1.3.1.21]
**Thupl.29377534s0013**	PANTHER	PTHR21257:SF38	-
**Thupl.29377763s0003**	PANTHER	PTHR10643	KINETOCHORE PROTEIN NDC80
**Thupl.29377763s0003**	PFAM	PF03801	HEC/Ndc80p family
**Thupl.29377763s0003**	KEGGORTH	K11547	kinetochore protein NDC80
**Thupl.29377763s0003**	KOG	KOG0995	Centromere-associated protein HEC1
**Thupl.29377905s0004**	PANTHER	PTHR11630:SF66	-
**Thupl.29377905s0004**	PFAM	PF00493	MCM2/3/5 family
**Thupl.29377905s0004**	GO	GO:0042555	MCM complex
**Thupl.29377905s0004**	GO	GO:0003678	DNA helicase activity
**Thupl.29377905s0004**	KOG	KOG0478	DNA replication licensing factor, MCM4 component
**Thupl.29377905s0004**	PFAM	PF17207	-
**Thupl.29377905s0004**	PFAM	PF14551	MCM N-terminal domain
**Thupl.29377905s0004**	KEGGORTH	K02212	minichromosome maintenance protein 4 (cell division control protein 54)
**Thupl.29377905s0004**	PANTHER	PTHR11630	DNA REPLICATION LICENSING FACTOR
**Thupl.29377905s0004**	GO	GO:0003677	DNA binding
**Thupl.29377905s0004**	EC	3.6.4.12	DNA helicase
**Thupl.29377905s0004**	GO	GO:0006270	DNA replication initiation
**Thupl.29377905s0004**	GO	GO:0005524	ATP binding
**Thupl.29378116s0023**	GO	GO:0016020	membrane
**Thupl.29378116s0023**	GO	GO:0015297	antiporter activity
**Thupl.29378116s0023**	PFAM	PF01554	MatE
**Thupl.29378116s0023**	GO	GO:0015238	drug transmembrane transporter activity
**Thupl.29378116s0023**	PANTHER	PTHR11206	MULTIDRUG RESISTANCE PROTEIN
**Thupl.29378116s0023**	GO	GO:0055085	transmembrane transport
**Thupl.29378116s0023**	KEGGORTH	K03327	multidrug resistance protein, MATE family
**Thupl.29378116s0023**	PANTHER	PTHR11206:SF85	-
**Thupl.29378116s0023**	KOG	KOG1347	Uncharacterized membrane protein, predicted efflux pump
**Thupl.29378116s0023**	GO	GO:0006855	drug transmembrane transport
**Thupl.29378169s0021**	PANTHER	PTHR36000	-
**Thupl.29378269s0005**	PANTHER	PTHR31391	FAMILY NOT NAMED
**Thupl.29378269s0005**	GO	GO:0003677	DNA binding
**Thupl.29378269s0005**	PFAM	PF02362	B3 DNA binding domain
**Thupl.29378329s0001**	PANTHER	PTHR31048:SF81	-
**Thupl.29378329s0001**	PFAM	PF00314	Thaumatin family
**Thupl.29378329s0001**	PANTHER	PTHR31048	FAMILY NOT NAMED
**Thupl.29378467s0019**	KOG	KOG2014	SMT3/SUMO-activating complex, AOS1/RAD31 component
**Thupl.29378467s0019**	PANTHER	PTHR10953	UBIQUITIN-ACTIVATING ENZYME E1

**Thupl.29378467s0019**	KEGGORTH	K10684	ubiquitin-like 1-activating enzyme E1 A [EC:6.3.2.19]
**Thupl.29378467s0019**	GO	GO:0008641	small protein activating enzyme activity
**Thupl.29378467s0019**	PANTHER	PTHR10953:SF162	-
**Thupl.29378467s0019**	PFAM	PF00899	ThiF family
**Thupl.29378604s0006**	NA	NA	NA
**Thupl.29378682s0038**	KEGGORTH	K00472	prolyl 4-hydroxylase [EC:1.14.11.2]
**Thupl.29378682s0038**	PFAM	PF13640	2OG-Fe(II) oxygenase superfamily
**Thupl.29378682s0038**	KOG	KOG1591	Prolyl 4-hydroxylase alpha subunit
**Thupl.29378682s0038**	PANTHER	PTHR10869:SF133	-
**Thupl.29378682s0038**	GO	GO:0055114	oxidation-reduction process
**Thupl.29378682s0038**	SIGNALP	SignalP-noTM	-
**Thupl.29378682s0038**	GO	GO:0016491	oxidoreductase activity
**Thupl.29378682s0038**	PANTHER	PTHR10869	PROLYL 4-HYDROXYLASE ALPHA SUBUNIT
**Thupl.29378682s0038**	EC	1.14.11.2	Procollagen-proline dioxygenase
**Thupl.29378750s0001**	PFAM	PF07847	Protein of unknown function (DUF1637)
**Thupl.29378750s0001**	PANTHER	PTHR22966:SF1	gb def: CG7550-PA
**Thupl.29378750s0001**	EC	1.13.11.19	Cysteamine dioxygenase
**Thupl.29378750s0001**	KOG	KOG4281	Uncharacterized conserved protein
**Thupl.29378750s0001**	GO	GO:0055114	oxidation-reduction process
**Thupl.29378750s0001**	KEGGORTH	K10712	cysteamine dioxygenase [EC:1.13.11.19]
**Thupl.29378750s0001**	GO	GO:0016702	oxidoreductase activity, acting on single donors with incorporation of molecular oxygen, incorporation of two atoms of oxygen
**Thupl.29378750s0001**	PANTHER	PTHR22966	UNCHARACTERIZED
**Thupl.29378953s0001**	PFAM	PF01535	PPR repeat
**Thupl.29378953s0001**	PANTHER	PTHR44149	-
**Thupl.29378953s0001**	PANTHER	PTHR44149:SF1	-
**Thupl.29378953s0001**	PFAM	PF13041	PPR repeat family
**Thupl.29378953s0001**	PFAM	PF12854	PPR repeat
**Thupl.29379280s0001**	PANTHER	PTHR22814:SF78	-
**Thupl.29379280s0001**	PFAM	PF00403	Heavy-metal-associated domain
**Thupl.29379280s0001**	GO	GO:0046872	metal ion binding
**Thupl.29379280s0001**	KOG	KOG1603	Copper chaperone
**Thupl.29379280s0001**	PANTHER	PTHR22814	COPPER TRANSPORT PROTEIN ATOX1-RELATED
**Thupl.29379280s0001**	GO	GO:0030001	metal ion transport
**Thupl.29379302s0001**	PANTHER	PTHR44566	-
**Thupl.29379302s0001**	GO	GO:0005515	protein binding
**Thupl.29379302s0001**	KOG	KOG0265	U5 snRNP-specific protein-like factor and related proteins
**Thupl.29379302s0001**	PFAM	PF00400	WD domain, G-beta repeat
**Thupl.29379321s0005**	KEGGORTH	K09838	zeaxanthin epoxidase [EC:1.14.13.90]

**Thupl.29379321s0005**	PANTHER	PTHR13789:SF245	-
**Thupl.29379321s0005**	PFAM	PF00498	FHA domain
**Thupl.29379321s0005**	KOG	KOG2614	Kynurenine 3-monooxygenase and related flavoprotein monooxygenases
**Thupl.29379321s0005**	EC	1.14.13.90	Zeaxanthin epoxidase
**Thupl.29379321s0005**	GO	GO:0005515	protein binding
**Thupl.29379321s0005**	PFAM	PF01494	FAD binding domain
**Thupl.29379321s0005**	GO	GO:0071949	FAD binding
**Thupl.29379321s0005**	PANTHER	PTHR13789	MONOOXYGENASE
**Thupl.29379402s0007**	PFAM	PF02668	Taurine catabolism dioxygenase TauD, TfdA family
**Thupl.29379402s0007**	GO	GO:0055114	oxidation-reduction process
**Thupl.29379402s0007**	PANTHER	PTHR10696	GAMMA-BUTYROBETAINE HYDROXYLASE-RELATED
**Thupl.29379402s0007**	PANTHER	PTHR10696:SF29	-
**Thupl.29379402s0007**	GO	GO:0016491	oxidoreductase activity
**Thupl.29379488s0007**	PFAM	PF13862	p21-C-terminal region-binding protein
**Thupl.29379488s0007**	KEGGORTH	K15262	-
**Thupl.29379488s0007**	PANTHER	PTHR13261	CDK INHIBITOR P21 BINDING PROTEIN
**Thupl.29379488s0007**	KOG	KOG3034	Isoamyl acetate-hydrolyzing esterase and related enzymes
**Thupl.29379722s0024**	PANTHER	PTHR10209:SF251	-
**Thupl.29379722s0024**	PANTHER	PTHR10209	OXIDOREDUCTASE, 2OG-FE(II) OXYGENASE FAMILY PROTEIN
**Thupl.29379722s0024**	PFAM	PF03171	2OG-Fe(II) oxygenase superfamily
**Thupl.29379722s0024**	GO	GO:0016491	oxidoreductase activity
**Thupl.29379722s0024**	PFAM	PF14226	non-haem dioxygenase in morphine synthesis N-terminal
**Thupl.29379722s0024**	EC	1.14.11.9	Flavanone 3-dioxygenase
**Thupl.29379722s0024**	KOG	KOG0143	Iron/ascorbate family oxidoreductases
**Thupl.29379722s0024**	GO	GO:0055114	oxidation-reduction process
**Thupl.29379747s0003**	PANTHER	PTHR33648	-
**Thupl.29379747s0003**	SIGNALP	SignalP-noTM	-
**Thupl.29379768s0005**	KEGGORTH	K19307	-
**Thupl.29379768s0005**	PANTHER	PTHR11538	PHENYLALANYL-TRNA SYNTHETASE
**Thupl.29379768s0005**	KOG	KOG4174	Uncharacterized conserved protein
**Thupl.29379768s0005**	PFAM	PF10354	Domain of unknown function (DUF2431)
**Thupl.29379768s0005**	PANTHER	PTHR11538:SF54	-
**Thupl.29379839s0006**	PANTHER	PTHR12169	ATPASE N2B
**Thupl.29379839s0006**	GO	GO:0005524	ATP binding
**Thupl.29379839s0006**	PANTHER	PTHR12169:SF6	-
**Thupl.29379839s0006**	EC	2.7.1.1	Hexokinase
**Thupl.29379933s0022**	GO	GO:0009247	glycolipid biosynthetic process

**Thupl.29379933s0022**	PFAM	PF04101	Glycosyltransferase family 28 C-terminal domain
**Thupl.29379933s0022**	GO	GO:0016758	transferase activity, transferring hexosyl groups
**Thupl.29379933s0022**	PFAM	PF06925	Monogalactosyldiacylglycerol (MGDG) synthase
**Thupl.29379933s0022**	EC	2.4.1.46	Monogalactosyldiacylglycerol synthase
**Thupl.29379933s0022**	PANTHER	PTHR43025	-
**Thupl.29379933s0022**	PANTHER	PTHR43025:SF1	-
**Thupl.29379933s0022**	KEGGORTH	K03715	1,2-diacylglycerol 3-beta-galactosyltransferase [EC:2.4.1.46]
**Thupl.29380045s0002**	PANTHER	PTHR23417:SF16	-
**Thupl.29380045s0002**	GO	GO:0008176	tRNA (guanine-N7-)-methyltransferase activity
**Thupl.29380045s0002**	KEGGORTH	K03439	tRNA (guanine-N7-)-methyltransferase [EC:2.1.1.33]
**Thupl.29380045s0002**	KOG	KOG3115	Methyltransferase-like protein
**Thupl.29380045s0002**	EC	2.1.1.33	tRNA (guanine(46)-N(7))-methyltransferase
**Thupl.29380045s0002**	PANTHER	PTHR23417	3-DEOXY-D-MANNO-OCTULOSONIC-ACID TRANSFERASE/TRNA (GUANINE-N(7)-)-METHYLTRANSFERASE
**Thupl.29380045s0002**	GO	GO:0006400	tRNA modification
**Thupl.29380045s0002**	PFAM	PF02390	Putative methyltransferase
**Thupl.29380174s0008**	PFAM	PF01195	Peptidyl-tRNA hydrolase
**Thupl.29380174s0008**	PANTHER	PTHR17224:SF4	-
**Thupl.29380174s0008**	SIGNALP	SignalP-noTM	-
**Thupl.29380174s0008**	PANTHER	PTHR17224	PEPTIDYL-TRNA HYDROLASE
**Thupl.29380174s0008**	KEGGORTH	K01056	peptidyl-tRNA hydrolase, PTH1 family [EC:3.1.1.29]
**Thupl.29380174s0008**	KOG	KOG2255	Peptidyl-tRNA hydrolase
**Thupl.29380174s0008**	EC	3.1.1.29	Aminoacyl-tRNA hydrolase
**Thupl.29380174s0008**	GO	GO:0004045	aminoacyl-tRNA hydrolase activity
**Thupl.29380345s0016**	GO	GO:0003723	RNA binding
**Thupl.29380345s0016**	PANTHER	PTHR17408:SF0	SUBFAMILY NOT NAMED
**Thupl.29380345s0016**	PANTHER	PTHR17408	HISTONE RNA HAIRPIN-BINDING PROTEIN
**Thupl.29380345s0016**	PFAM	PF15247	Histone RNA hairpin-binding protein RNA-binding domain
**Thupl.29380345s0016**	GO	GO:0003729	mRNA binding
**Thupl.29380839s0001**	SIGNALP	SignalP-TM	-
**Thupl.29380839s0001**	PANTHER	PTHR34132:SF1	-
**Thupl.29380839s0001**	PANTHER	PTHR34132	-
**Thupl.29380857s0017**	PANTHER	PTHR16019:SF13	-
**Thupl.29380857s0017**	PFAM	PF03909	BSD domain
**Thupl.29380857s0017**	PANTHER	PTHR16019	SYNAPSE-ASSOCIATED PROTEIN
**Thupl.29380857s0017**	KOG	KOG2690	Uncharacterized conserved protein, contains BSD domain
**Thupl.29380857s0020**	PANTHER	PTHR31793:SF24	-

**Thupl.29380857s0020**	PANTHER	PTHR31793	FAMILY NOT NAMED
**Thupl.29380857s0020**	PFAM	PF03061	Thioesterase superfamily
**Thupl.29380989s0001**	EC	2.4.2.30	NAD(+) ADP-ribosyltransferase
**Thupl.29380989s0001**	PFAM	PF00644	Poly(ADP-ribose) polymerase catalytic domain
**Thupl.29380989s0001**	PANTHER	PTHR21328:SF2	POLY (ADP-RIBOSE) POLYMERASE FAMILY, MEMBER 6 (PARP6)
**Thupl.29380989s0001**	GO	GO:0003950	NAD+ ADP-ribosyltransferase activity
**Thupl.29380989s0001**	PANTHER	PTHR21328	POLY (ADP-RIBOSE) POLYMERASE FAMILY, MEMBER (PARP)
**Thupl.29381152s0001**	PANTHER	PTHR12458:SF8	-
**Thupl.29381152s0001**	KOG	KOG3213	Transcription factor IIB
**Thupl.29381152s0001**	PANTHER	PTHR12458	ORF PROTEIN
**Thupl.29381152s0001**	PFAM	PF05018	Protein of unknown function (DUF667)
**Thupl.29381153s0002**	PANTHER	PTHR33384	-
**Thupl.29381693s0002**	GO	GO:0004553	hydrolase activity, hydrolyzing O-glycosyl compounds
**Thupl.29381693s0002**	PFAM	PF00332	Glycosyl hydrolases family 17
**Thupl.29381693s0002**	EC	3.2.1.39	Glucan endo-1,3-beta-D-glucosidase
**Thupl.29381693s0002**	SIGNALP	SignalP-noTM	-
**Thupl.29381693s0002**	PANTHER	PTHR32227	FAMILY NOT NAMED
**Thupl.29381693s0002**	GO	GO:0005975	carbohydrate metabolic process
**Thupl.29381693s0002**	PANTHER	PTHR32227:SF72	-
**Thupl.29381804s0017**	PFAM	PF09493	Tryptophan-rich protein (DUF2389)
**Thupl.29382099s0019**	PANTHER	PTHR35302	-
**Thupl.29382099s0019**	PFAM	PF12046	Protein of unknown function (DUF3529)
**Thupl.29382102s0017**	NA	NA	NA
**Thupl.29382330s0004**	EC	2.5.1.75	tRNA dimethylallyltransferase
**Thupl.29382330s0004**	KEGGORTH	K00791	tRNA dimethylallyltransferase [EC:2.5.1.75]
**Thupl.29382330s0004**	KOG	KOG1384	tRNA delta(2)-isopentenylpyrophosphate transferase
**Thupl.29382330s0004**	PANTHER	PTHR11088	TRNA DELTA(2)-ISOPENTENYLPYROPHOSPHATE TRANSFERASE-RELATED
**Thupl.29382330s0004**	PFAM	PF01715	IPP transferase
**Thupl.29382330s0004**	PANTHER	PTHR11088:SF50	-
**Thupl.29382330s0004**	GO	GO:0008033	tRNA processing
**Thupl.29382473s0007**	KOG	KOG1746	Defender against cell death protein/oligosaccharyltransferase, epsilon subunit
**Thupl.29382473s0007**	EC	2.4.99.18	Dolichyl-diphosphooligosaccharide--protein glycotransferase
**Thupl.29382473s0007**	GO	GO:0008250	oligosaccharyltransferase complex
**Thupl.29382473s0007**	PFAM	PF02109	DAD family

**Thupl.29382473s0007**	KEGGORTH	K12668	oligosaccharyltransferase complex subunit epsilon
**Thupl.29382473s0007**	GO	GO:0004579	dolichyl-diphosphooligosaccharide-protein glycotransferase activity
**Thupl.29382473s0007**	PANTHER	PTHR10705	DOLICHYL-DIPHOSPHOOLIGOSACCHARIDE--PROTEIN GLYCOSYLTRANSFERASE SUBUNIT DAD1
**Thupl.29382473s0007**	GO	GO:0016021	integral to membrane

Table S11. Kruskal-Wallis analysis of variance in π and d_{XY} estimates in single-copy orthologs across subpopulations.

Subpopulation	Average π (SD)	Kruskal-Wallis χ^2	*p*-value
Northern-Coastal	0.00267 (0.0120)	5.79	0.0554
Central	0.00267 (0.0119)		
Southern-Interior	0.00266 (0.0135)		
**Subpop. comparison**	**Mean d_{XY} (SD)**	**Kruskal-Wallis χ^2**	***p*-value**
Northern-Coastal/Central	0.00272 (0.0130)	2.12	0.347
Northern-Coastal/Southern-Interior	0.00277 (0.0123)		
Central/Southern-Interior	0.00280 (0.0127)		

Table S12. Mean (SD) observed heterozygosity (H) and inbreeding coefficient (*F*) in the range-wide population (RWP) (*n* = 112) and each generation of selfing lines (FS – S4: *n* = 28; S5: *n* = 11).

	H	*F*
**RWP**	0.219 (0.110)	0.331 (0.181)
**FS**	0.296 (0.174)	0.00569 (0.0312)
**S1**	0.148 (0.103)	0.428 (0.0610)
**S2**	0.0777 (0.0659)	0.616 (0.0678)
**S3**	0.0328 (0.0422)	0.724 (0.0785)
**S4**	0.0194 (0.0314)	0.789 (0.0669)
**S5**	0.00786 (0.0281)	0.801 (0.0733)

Table S13. A) Pairwise Sign test for variance of median observed (H_O) and expected (H_E) heterozygosities in each generation of selfing lines (SLs), testing whether median H_O is significantly lower than median H_E. B) One-tailed Wilcoxon signed rank test for variance of mean H_O and H_E in each generation of SLs, testing whether mean H_O is significantly lower than mean H_E. C) One-sample *t*-test for variance of mean observed (F_O) and expected (F_E) inbreeding coefficients in each generation of SLs.

A

Generation	H_O (median)	H_E (median)	*S*	*p*-value
FS	0.296	-	-	-
S1	0.143	0.148	7338	0.0710
S2	0.0714	0.0741	7843	0.000223
S3	0.0357	0.0370	6072	$< 2.2\times10^{-16}$
S4	0	0.0185	6641	$< 2.2\times10^{-16}$
S5	0	0.00926	4544	$< 2.2\times10^{-16}$

B

Generation	H_O (mean)	H_E (mean)	*V*	*p*-value
FS	0.296	-	-	-
S1	0.148	0.148	54445812	0.0803
S2	0.0777	0.0741	68311222	1
S3	0.0328	0.0371	53919636	$< 2.2\times10^{-16}$
S4	0.0194	0.0185	65921119	$< 2.2\times10^{-16}$
S5	0.00786	0.00926	23642536	$< 2.2\times10^{-16}$

C

Generation	F_O (mean)	F_E (mean)	*t*	95% CI	*p*-value
FS	0.00569	0	0.966	-0.00640, 0.0178	0.343
S1	0.428	0.5	-6.24	0.404, 0.452	1.12×10^{-6}
S2	0.616	0.75	-10.5	0.590, 0.642	5.29×10^{-11}
S3	0.724	0.875	-10.1	0.694, 0.755	1.05×10^{-10}
S4	0.789	0.9375	-11.7	0.763, 0.815	4.29×10^{-12}
S5	0.801	0.96875	-7.59	0.752, 0.850	1.87×10^{-5}

Table S14. Gene Ontology (GO) categories for genes containing outlier SNPs (*n* = 83).

	GO category	GO ID	# of genes with GO category	Frequency (out of 13 genes)
*	GO:0016021	integral to membrane	3	0.230769
	GO:0006355	regulation of transcription, DNA-dependent	2	0.153846
	GO:0000287	magnesium ion binding	1	0.076923
*	GO:0003690	double-stranded DNA binding	1	0.076923
	GO:0003700	sequence-specific DNA binding transcription factor activity	1	0.076923
*	GO:0004012	phospholipid-translocating ATPase activity	1	0.076923
	GO:0004252	serine-type endopeptidase activity	1	0.076923
*	GO:0004518	nuclease activity	1	0.076923
	GO:0005515	protein binding	1	0.076923
	GO:0005524	ATP binding	1	0.076923
	GO:0005525	GTP binding	1	0.076923
*	GO:0005743	mitochondrial inner membrane	1	0.076923
	GO:0006281	DNA repair	1	0.076923
	GO:0006508	proteolysis	1	0.076923
	GO:0006629	lipid metabolic process	1	0.076923
*	GO:0006850	mitochondrial pyruvate transport	1	0.076923
	GO:0007165	signal transduction	1	0.076923
	GO:0008270	zinc ion binding	1	0.076923
*	GO:0010112	regulation of systemic acquired resistance	1	0.076923
*	GO:0015095	magnesium ion transmembrane transporter activity	1	0.076923
*	GO:0015693	magnesium ion transport	1	0.076923
*	GO:0015914	phospholipid transport	1	0.076923
	GO:0016491	oxidoreductase activity	1	0.076923
	GO:0043531	ADP binding	1	0.076923
	GO:0043565	sequence-specific DNA binding	1	0.076923
	GO:0055085	transmembrane transport	1	0.076923
	GO:0055114	oxidation-reduction process	1	0.076923

* Over-represented in outlier SNPS when compared to the full SNP set (*n* = 18,371) ($p < 0.05$, Fisher's Exact Test)

Table S15. Geographic locations of origin of parent trees for selfing lines.

Parent	Selfing Line	Location	Latitude (° N)	Longitude (° W)	Elevation (m)
491	1	Bacchante Bay	49.45	-126.10	305
13	1	O'Connell Lake	50.35	-127.70	122
150	6	Tzoonie River	49.87	-123.73	450
49	6	Misty Lake	50.63	-127.28	91
213	7	Norrish Creek	49.22	-122.15	400
76	7	Hathaway Creek	50.58	-127.82	105
142	8	Bedwell Sound	49.37	-125.75	490
289	8	Misty Lake	50.63	-127.30	60
263	10	Narrows Inlet	49.08	-123.73	120
306	10	Denad Creek	50.55	-128.00	25
341	13	Utlah Creek	50.28	-127.43	183
323	13	Koprino River	50.55	-127.85	280
367	16	Henderson Lake	49.10	-125.02	200
381	16	Sydney Inlet	49.48	-126.28	65
386	17	Cypre River	49.37	-125.87	180
253	17	Labouchere Channel	52.03	-127.25	152
403	19	Eelstow Passage	50.10	-127.17	60
191	19	Jervis Inlet	50.12	-123.78	98
424	20	Chatham Channel	50.52	-126.25	75
318	20	Ronning Creek	50.57	-128.27	105
435	21	Chemainus	48.93	-123.75	115
15	21	Mahatta Creek	50.40	-127.78	155
461	23	Artlish Caves	50.13	-127.08	100
5	23	Mahatta Creek	50.40	-127.78	122
344	26	Victoria Lake	50.05	-127.42	457
313	26	Brink Lake	50.68	-128.27	213
343	27	Victoria Lake	50.05	-127.42	457
319	27	Ronning Creek	50.57	-128.27	115
333	29	Marble River	50.25	-127.33	244
312	29	Brink Lake	50.70	-128.27	122

Supplemental Datasets

Dataset S1. Primary transcripts in FASTA format.

Dataset S2. Predicted gene functions for annotated genes.

Dataset S3. List of western redcedar (WRC) single-copy ortholog (SCO) genes.

Dataset S4. Sequences used for validation of the genome annotation in FASTA format.

Dataset S5. Filtered SNP genotypes for the range-wide population (RWP) in VCF format (*n* = 18,371 SNPs).

Dataset S6. VEP SNP annotation for all annotated SNPS.

Dataset S7. WRC SNPs successfully mapped to putative linkage groups (LGs) on the *Sequoiadendron giganteum* genome (*n* = 26,140 SNPs).

Dataset S8. Corrected SNP genotypes for manually corrected and imputed selfing lines in PLINK binary format (*n* = 18,371 SNPs).

Dataset S9. Statistical analysis and outcomes for SNP fate based on expectations of genetic drift for all SNPs after four generations of selfing.

Dataset S10. Annotation of outlier SNPs deviating from expectations of genetic drift during selfing.

Supplemental Code

Code S1. Geographic map used in Figure 1.

Code S2. F_{st} analysis, DAPC, PCA plots used in Figure 1.

Code S3. LD plots used in Figure 1. Code derived and adapted from a post made here: https://www.biostars.org/p/347796/.

Code S4. Analysis of pi, dxy in pixy, plots used in Figure 3.

Code S5. Manual correction of SNP data for complete selfing lines. Further fine-scale manual correction was carried out by hand.

Code S6. Analysis of heterozygosity, statistical tests, chi-squared test for analysis of loci under selection during selfing (outlier SNPs), plots used in Figure 4.

Code S7. Analysis and plots for inbreeding coefficients in Figure 4.

Code S8. Analysis of outlier SNPs during selfing, variant effects, statistical analyses of SNP consequences and GO categories, annotation of SNPs.

SI References

Birol I, Raymond A, Jackman SD, Pleasance S, Coope R, Taylor GA, Yuen MM, Keeling CI, Brand D, Vandervalk BP, et al. 2013. Assembling the 20 Gb white spruce (*Picea glauca*) genome from whole-genome shotgun sequencing data. *Bioinformatics* **29**: 1492–1497. doi: 10.1093/bioinformatics/btt178.

Garrison E, Marth G. 2012. Haplotype-based variant detection from short-read sequencing. *arXiv Prepr* **1207.3907**. http://arxiv.org/abs/1207.3907 (Accessed April 9, 2021).

Haas BJ, Delcher AL, Mount SM, Wortman JR, Smith RK, Hannick LI, Maiti R, Ronning CM, Rusch DB, Town CD, et al. 2003. Improving the *Arabidopsis* genome annotation using maximal transcript alignment assemblies. *Nucleic Acids Res* **31**: 5654–5666. doi: 10.1093/nar/gkg770.

Jackman SD, Coombe L, Chu J, Warren RL, Vandervalk BP, Yeo S, Xue Z, Mohamadi H, Bohlmann J, Jones SJM, et al. 2018. Tigmint: Correcting assembly errors using linked reads from large molecules. *BMC Bioinformatics* **19** doi: 10.1186/s12859-018-2425-6.

Jackman SD, Vandervalk BP, Mohamadi H, Chu J, Yeo S, Hammond SA, Jahesh G, Khan H, Coombe L, Warren RL, et al. 2017. ABySS 2.0: Resource-efficient assembly of large genomes using a Bloom filter. *Genome Res* **27**: 768–777. doi: 10.1101/gr.214346.116.

Lander ES, Waterman MS. 1988. Genomic mapping by fingerprinting random clones: A mathematical analysis. *Genomics* **2**: 231–239. doi: 10.1016/0888-7543(88)90007-9.

Li H. 2013. Aligning sequence reads, clone sequences and assembly contigs with BWA-MEM. *arXiv Prepr* **1303.3997**. http://arxiv.org/abs/1303.3997 (Accessed April 9, 2021).

Liu X, Fu Y-X. 2020. Stairway Plot 2: demographic history inference with folded SNP frequency spectra. *Genome Biol* **21**: 280. doi: 10.1186/s13059-020-02196-9.

McLaren W, Gil L, Hunt SE, Riat HS, Ritchie GRS, Thormann A, Flicek P, Cunningham F. 2016. The Ensembl Variant Effect Predictor. *Genome Biol* **17** doi: 10.1186/s13059-016-0974-4.

Miller JM, Cullingham CI, Peery RM. 2020. The influence of *a priori* grouping on inference of genetic clusters: simulation study and literature review of the DAPC method. *Heredity (Edinb)* **125** doi: 10.1038/s41437-020-0348-2.

Mohamadi H, Khan H, Birol I. 2017. ntCard: A streaming algorithm for cardinality estimation in genomics data. *Bioinformatics* **33**: 1324–1330. doi: 10.1093/bioinformatics/btw832.

Neale DB, McGuire PE, Wheeler NC, Stevens KA, Crepeau MW, Cardeno C, Zimin AV, Puiu D, Pertea GM, Sezen UU, et al. 2017. The Douglas-Fir genome sequence reveals specialization of the photosynthetic apparatus in Pinaceae. *G3 Genes, Genomes, Genet* **7**: 3157–3167. doi: 10.1534/g3.117.300078.

Nystedt B, Street NR, Wetterbom A, Zuccolo A, Lin YC, Scofield DG, Vezzi F, Delhomme N, Giacomello S, Alexeyenko A, et al. 2013. The Norway spruce genome sequence and conifer genome evolution. *Nature* **497**: 579–584. doi: 10.1038/nature12211.

O'Connell J, Schulz-Trieglaff O, Carlson E, Hims MM, Gormley NA, Cox AJ. 2015. NxTrim: Optimized trimming of Illumina mate pair reads. *Bioinformatics* **31**: 2035–2037. doi: 10.1093/bioinformatics/btv057.

O'Connell LM, Ritland K, Thompson SL. 2008. Patterns of post-glacial colonization by western redcedar (*Thuja plicata*, Cupressaceae) as revealed by microsatellite markers. *Botany* **86**: 194–203. doi: 10.1139/B07-124.

Salamov AA, Solovyev VV. 2000. *Ab initio* gene finding in *Drosophila* genomic DNA. *Genome Res* **10**: 516–522. doi: 10.1101/gr.10.4.516.

Scott AD, Zimin AV, Puiu D, Workman R, Britton M, Zaman S, Caballero M, Read AC, Bogdanove AJ, Burns E, et al. 2020. A reference genome sequence for giant sequoia. *G3 Genes, Genomes, Genet* **10**: 3907–3919. doi: 10.1534/g3.120.401612.

Shalev TJ, Yuen MMS, Gesell A, Yuen A, Russell JH, Bohlmann J. 2018. An annotated transcriptome of highly inbred *Thuja plicata* (Cupressaceae) and its utility for gene discovery of terpenoid biosynthesis and conifer defense. *Tree Genet Genomes* **14** doi: 10.1007/s11295-018-1248-y.

Shengqiang S, Goodstein D, Rokhsar D. 2013. PERTRAN: Genome-guided RNA-seq Read Assembler. In *Cold Spring Harbor Lab Genome Informatics*, New York, NY.

Slater GSC, Birney E. 2005. Automated generation of heuristics for biological sequence comparison. *BMC Bioinformatics* **6** doi: 10.1186/1471-2105-6-31.

Smit A, Hubley R. 2015. RepeatModeler open-1.0. 2008-2015. *http://www.repeatmasker.org*. http://www.repeatmasker.org.

Smit A, Hubley R, Green P. 2015. RepeatMasker Open-4.0. 2013-2015 . *http://www.repeatmasker.org*. http://www.repeatmasker.org.

Stevens KA, Wegrzyn JL, Zimin A, Puiu D, Crepeau M, Cardeno C, Paul R, Gonzalez-Ibeas D, Koriabine M, Holtz-Morris AE, et al. 2016. Sequence of the sugar pine megagenome. *Genetics* **204**: 1613–1626. doi: 10.1534/genetics.116.193227.

Vurture GW, Sedlazeck FJ, Nattestad M, Underwood CJ, Fang H, Gurtowski J, Schatz MC. 2017. GenomeScope: Fast reference-free genome profiling from short reads. *Bioinformatics* **33**: 2202–2204. doi: 10.1093/bioinformatics/btx153.

Warren RL, Keeling CI, Yuen MM Saint, Raymond A, Taylor GA, Vandervalk BP, Mohamadi H, Paulino D, Chiu R, Jackman SD, et al. 2015. Improved white spruce (*Picea glauca*) genome assemblies and

annotation of large gene families of conifer terpenoid and phenolic defense metabolism. *Plant J* **83**: 189–212. doi: 10.1111/tpj.12886.

Weir BS, Cockerham CC. 1984. Estimating F-Statistics for the Analysis of Population Structure. *Evolution* **38**: 1358–1370. doi: 10.2307/2408641.

Wu TD, Nacu S. 2010. Fast and SNP-tolerant detection of complex variants and splicing in short reads. *Bioinformatics* **26** doi: 10.1093/bioinformatics/btq057.

Xin Z, Chen J. 2012. A high throughput DNA extraction method with high yield and quality. *Plant Methods* **8** doi: 10.1186/1746-4811-8-26.

Yeo S, Coombe L, Warren RL, Chu J, Birol I. 2018. ARCS: Scaffolding genome drafts with linked reads. *Bioinformatics* **34**: 725–731. doi: 10.1093/bioinformatics/btx675.

Zimin A, Stevens KA, Crepeau MW, Holtz-Morris A, Koriabine M, Marçais G, Puiu D, Roberts M, Wegrzyn JL, de Jong PJ, et al. 2014. Sequencing and assembly of the 22-Gb loblolly pine genome. *Genetics* **196**: 875–890. doi: 10.1534/genetics.113.159715.
